# Potential for prolonged replication of common acute respiratory viruses in air-liquid interface cultures of primary human airway cells

**DOI:** 10.1128/msphere.00422-25

**Published:** 2025-08-28

**Authors:** Miyuki Kawase, Reiko Suwa, Satoko Sugimoto, Masatoshi Kakizaki, Yohei Kume, Hisao Okabe, Sakurako Norito, Makoto Ujike, Hayato Go, Mitsuaki Hosoya, Koichi Hashimoto, Kazuya Shirato

**Affiliations:** 1 Department of Virology III, National Institute of Infectious Disease, Musashimurayama, Tokyo, Japan; 2 Department of Respiratory Viruses, National Institute of Infectious Diseases, Japan Institute for Health Security 13511, Musashimurayama, Tokyo, Japan; 3 Management Department Research Center for Biosafety, Laboratory Animals, and Pathogen Bank, National Institute of Infectious Diseases 13511, Musashimurayama, Tokyo, Japan; 4 Department of Pediatrics, School of Medicine, Fukushima Medical University 714257, Fukushima, Fukushima, Japan; 5 Faculty of Veterinary Medicine, Nippon Veterinary and Life Science University 12989, Musashino, Tokyo, Japan; 6 Department of Perinatology and Pediatrics for Regional Medical Support, Fukushima Medical University 12775, Fukushima, Fukushima, Japan; Johns Hopkins University Bloomberg School of Public Health, Baltimore, Maryland, USA

**Keywords:** respiratory viruses, replication, HBTEC-ALI, HNEpC-ALI

## Abstract

A previous study provides clinical evidence that extended respiratory virus detection can occur in healthy pediatric individuals. However, it remains unclear how long respiratory viruses can survive in the human respiratory tissue. In this study, the replication potential of common respiratory viruses was evaluated using air-liquid interface (ALI) cultures of primary human respiratory epithelial cells. The findings demonstrate that most respiratory viruses can replicate for approximately 100 days in ALI cultures, with some showing prolonged replication for up to 150–200 days. In contrast, influenza and respiratory DNA viruses showed shorter replication periods likely due to virus-induced cell death. Even during the late phase, some samples continued to support viral replication in re-infection experiments, indicating sustained viral viability. ALI cultures, which lack effector immune cells, did not exhibit apparent type I interferon responses during long-term replication, except for transient IFNβ secretion in the early phase of infection, suggesting a state of tolerance that allows prolonged viral replication. Furthermore, genetic analysis revealed that viruses replicating for more than 50–60 days developed genetic variations, indicating an increased risk of mutations during prolonged infection. These results suggest that common respiratory viruses can remain detectable in human respiratory tissues for extended periods. However, transmission within 50–60 days may be preferable to reduce the risk of generating genetic variants. This concern is particularly relevant in immunocompromised individuals, where prolonged infections may facilitate viral evolution and contribute to the emergence of novel variants.

**IMPORTANCE:**

This study demonstrates that most common respiratory viruses, excluding influenza and DNA viruses, can replicate and produce infectious progeny for an average of up to 100 days in air-liquid interface (ALI) cultures of primary human respiratory epithelial cells without obvious innate immune responses. These findings imply that extended viral replication may occur in human hosts, potentially supported by the slow turnover of the respiratory epithelium. Notably, replication beyond 50–60 days was associated with the accumulation of genetic variations, suggesting a potential mechanism for the emergence of novel variants. To mitigate this risk, limiting transmission to within 50–60 days may be preferable. This issue is particularly relevant in immunocompromised individuals, where prolonged infection may promote viral evolution. Together, these findings provide insight into the replication dynamics of respiratory viruses in human tissue and highlight the importance of limiting long-term replication to prevent the emergence of new variants.

## INTRODUCTION

Acute respiratory infections (ARIs) are the leading cause of mortality in children worldwide (1), and a wide range of bacterial, viral, and fungal pathogens have been implicated in their development (2–4).

The coronavirus disease 2019 (COVID-19) pandemic caused by severe acute respiratory syndrome coronavirus 2 (SARS-CoV-2), which emerged in late 2019, disrupted the seasonal dynamics of pre-existing respiratory viruses (5–7). In Japan, the incidence of enveloped viruses—readily inactivated by alcohol-based disinfectants—declined markedly in 2020 due to enhanced hygiene measures, while non-enveloped viruses continued to be detected at similar levels as before the pandemic (5–7). Respiratory syncytial virus (RSV) and human parainfluenzavirus (HPIV) were detected again in 2021, followed by influenza viruses and human metapneumovirus (hMPV) in 2022 (5–7). Notably, RSV and hMPV isolates detected post-pandemic were genetically similar to viruses circulating prior to the COVID-19 outbreak (8, 9), raising important questions about the mechanisms by which these viruses were maintained during the non-epidemic periods.

Owusu et al. reported that human coronavirus (HCoV) genomes were detectable in specimens from healthy adults, with no significant differences between symptomatic and asymptomatic individuals (10). These findings suggest that HCoVs may circulate broadly in the population year-round, independent of symptoms or seasonality. Although similar studies are lacking for other respiratory viruses, it is plausible that they too are maintained in asymptomatic individuals. To investigate this, longitudinal sampling and repeated virus detection in human populations are required. Recently, Teoh et al. conducted a longitudinal cohort study involving nasal specimen collection from healthy children (11). Their findings showed that although the median duration of viral detection was 1–2 weeks, prolonged detection occurred in 23.4% of all respiratory virus infections. Specifically, HBoV1, HRV, and ADV were detectable for 14 to 39 weeks, while HCoV-NL63, OC43, RSV, hMPV, and respiroviruses were detectable for up to 4 to 6 weeks. Notably, RSV was sometimes detected over prolonged periods, even in asymptomatic individuals. Their report provides clinical evidence that extended respiratory virus detection can occur in healthy pediatric populations; however, there is a question of how long respiratory viruses can survive in the human respiratory tissue.

In this study, we investigated the replication potential of common respiratory viruses in the human respiratory tract using clinical isolates. Air-liquid interface (ALI) cultures of primary human respiratory epithelial cells represent a robust *ex vivo* system for culturing various respiratory viruses (12–14). These cultures comprise differentiated epithelial cells devoid of immune components, making them particularly well suited for evaluating the duration of virus replication in respiratory tissues. In this study, we employed ALI cultures derived from human bronchial/tracheal epithelial cells (HBTEC) and primary human nasal epithelial cells (HNEpC) to assess the long-term replication capacity of a panel of clinical respiratory virus isolates.

## RESULTS

### Ongoing replication of common respiratory viruses in HBTEC-ALI and HNEpC-ALI cultures

**Fig 1:**
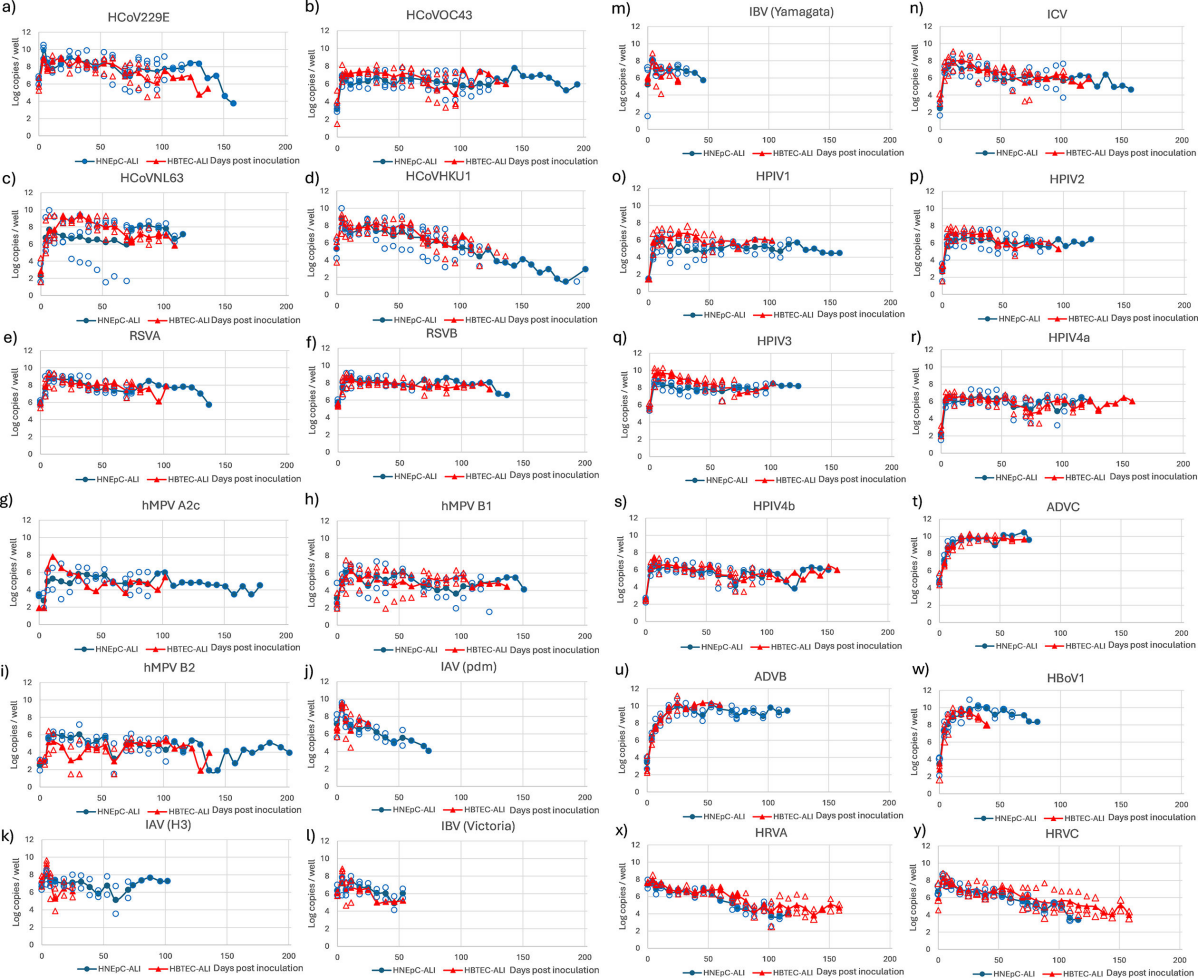
Ongoing replication of common respiratory viruses in HBTEC-ALI and HNEpC-ALI cultures. Clinical isolates of respiratory viruses were inoculated onto ALI cultures, and cell washes were collected approximately every 7 days post-inoculation. Viral replication was monitored by real-time RT-PCR. The viruses tested include: (**a**) HCoV-229E, (**b**) HCoV-OC43, (**c**) HCoV-NL63, (**d**) HCoV-HKU1, (**e**) RSV-A, (**f**) RSV-B, (**g**) hMPV A2c, (**h**) hMPV B1, (**i**) hMPV B2, (**j**) IAV (pdm), (**k**) IAV (**H3**), (**l**) IAV (Victoria), (**m**) IBV (Yamagata), (**n**) ICV, (**o**) HPIV-1, (**p**) HPIV-2, (**q**) HPIV-3, (**r**) HPIV-4a, (**s**) HPIV-4b, (**t**) ADV-C, (**u**) ADV-B3, (**w**) HBoV-1, (**x**) HRV-A, and (**y**) HRV-C. Red lines and triangles represent data from HBTEC-ALI cultures; blue lines and triangles represent data from HNEpC-ALI cultures. Open symbols indicate individual data points (*n* = 3); closed symbols represent average values.

**Fig 2:**
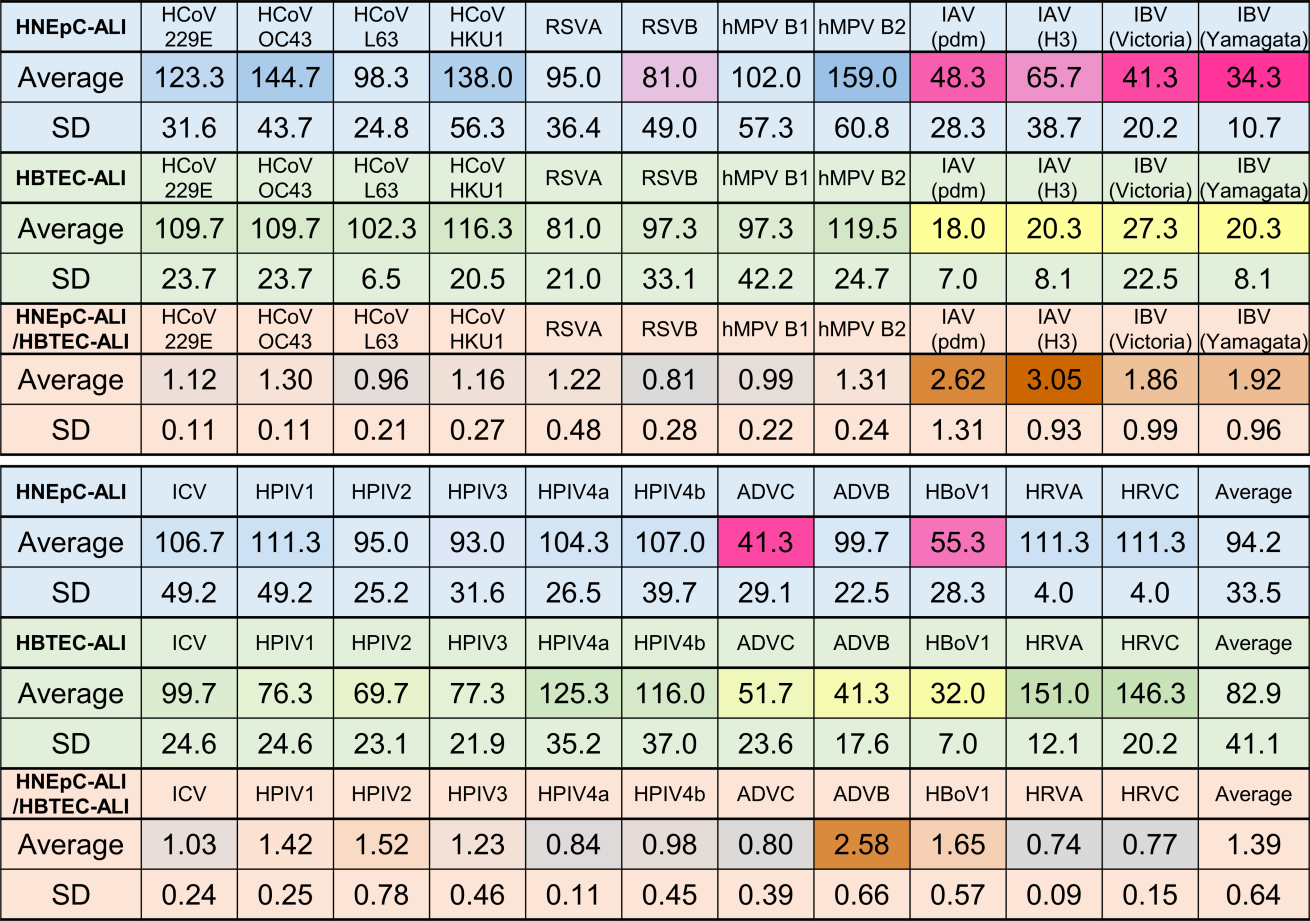
Summary of the duration of the respiratory virus replication in ALI cultures. The top and middle panels show the average duration and standard deviation of virus replication in HNEpC-ALI and HBTEC-ALI cultures, respectively. Shading indicates deviation from the overall average replication duration: pink (HNEpC-ALI) and cream-yellow (HBTEC-ALI) denote statistically significant differences. The bottom panel shows the relative duration of replication in HNEpC-ALI compared to HBTEC-ALI. Dark brown shading indicates a statistically significant difference from the overall mean.

To evaluate the replication potential of common respiratory viruses, 24 isolates representing 14 viral species were inoculated onto the HBTEC-ALI and HNEpC-ALI cultures. Viral replication was monitored by real-time RT-PCR using apical cell washes collected every 7 days post-inoculation (Fig. 1). The average duration of replication is summarized in Fig. 2. HCoVs replicated for more than 100 days on average, with prolonged replication particularly evident in HNEpC-ALI cultures, except for HCoV-NL63 (Fig. 1a through d and 2). Pneumoviruses also replicated for approximately 100 days on average (Fig. 1e through i and 2), with certain isolates, including HCoV-OC43, HCoV-HKU1, and hMPV-B2, showing replication for nearly 200 days. Influenza A and B viruses (IAVs and IBVs) replicated for 18.0–27.3 days in HBTEC-ALI and 34.3–65.7 days in HNEpC-ALI, significantly shorter than other virus groups (Fig. 1j through m and 2). In contrast, influenza C virus (ICV) replicated for approximately 100 days (Fig. 1n and 2). HPIVs replicated for around 100 days in HNEpC-ALI cultures; in HBTEC-ALI, HPIV1–3 replicated for 69.7–77.3 days, whereas HPIV4 showed longer replication durations (116.0–125.3 days) (Fig. 2). DNA viruses (adenovirus C [ADVC], adenovirus B3 [ADVB3), and human bocavirus 1 [HBoV1]) generally exhibited shorter replication periods (32.0–51.7 days in HBTEC-ALI and 41.3–55.3 days in HNEpC-ALI), except for ADVB3, which replicated for approximately 100 days in HNEpC-ALI (Fig. 1t through w and 2). Human rhinoviruses (HRVs) replicated for 111.3 days in HNEpC-ALI and for 146.3–151.0 days in HBTEC-ALI (Fig. 1x and y and 2). Influenza and DNA viruses induced cell death as a termination point of replication, whereas other viruses generally did not cause cytopathic effects (CPEs), particularly after replication became undetectable. In viruses showing long-term replication, viral copy numbers remained stable or gradually declined over time. Overall, the average replication durations for the respiratory viruses tested were 94.2 days in HNEpC-ALI and 82.9 days in HBTEC-ALI, indicating that many respiratory viruses can remain replicative in human airway cells for approximately 3 months, with the exception of influenza and DNA viruses (Fig. 2). Notably, both IAV and ADVB3 replicated significantly longer in HNEpC-ALI than in HBTEC-ALI (*P* < 0.05) (Fig. 2).

### Infectivity of long-cultured viruses in human airway ALI cultures

Most common respiratory viruses were maintained for several weeks, and some continued to replicate for 150 to 200 days. To determine whether viruses present during extended culture remained infectious, the presence of infectious virus was assessed across three defined phases of replication: early (7–11 days), middle (18–60 days), and late (67–202 days), with the late phase further subdivided based on strain-specific replication durations (Table 1). Virus isolation of pneumoviruses is generally challenging due to their thermolability. Recent studies have shown that fusion proteins of circulating RSV strains are prone to inactivation at low temperatures (15, 16), which may have contributed to the low recovery rate following sample freezing. Nevertheless, infectious virus was detected in samples collected not only during early and middle phases but also during late-phase replication. To minimize potential biases due to viral adaptation or differing susceptibility of host cells, real-time RT-PCR was used as a standardized method to assess viral replication across all clinical isolates. Samples that supported viral replication during re-infection experiments exhibited sufficient viral copy numbers, indicating the retention of infectivity, even after extended incubation. These results suggest that infectious virus production can continue for several weeks and, in some cases, up to 150 days. Re-infection success rates were lower in late-phase samples compared to early and middle phases. In particular, HCoV-NL63, HCoV-HKU1, HBoV1, HRV-A, and HRV-C showed significantly reduced infectivity in the late phase (*P* < 0.05), suggesting a potential for latency or reduced replication competence. Additionally, HCoV-HKU1, ICV, HPIV1, and HPIV4 demonstrated higher re-infection success in HBTEC-ALI than in HNEpC-ALI (*P* < 0.05), indicating a possible preference for bronchial/tracheal epithelial cells in sustaining viral propagation.

**TABLE 1:** Re-infection of long-term cultured viruses in HBTEC-ALI and HNEpC-ALI cultures

		Early	Middle	Late		
				1	2	3
HCoV229E	Days	7 days	53 days	96–116 days	137–158 days	N/A^*b*^
	HNEpC-ALI	2/3^*a*^	2/3	1/3	0/1	N/A
	Copies^*c*^	8.3	9.0	8.2	N/A	N/A
	HBTEC-ALI	3/3	3/3	3/3	0/1	N/A
	Copies	7.8	8.5	6.7	N/A	N/A
HCoVOC43	Days	7 days	53 days	96–116 days	123–158 days	195 days
	HNEpC-ALI	3/3	2/3	2/3	1/2	1/1
	Copies	5.5	5.2	4.8	6.1	5.3
	HBTEC-ALI	3/3	3/3	2/3	1/1	N/A
	Copies	6.7	6.6	6.6	8.1	N/A
HCoVNL63	Days	7 days	46–53 days	67–116 days	N/A	N/A
	HNEpC-ALI	1/3	2/3	0/3	N/A	N/A
	Copies	7.5	4.7	N/A	N/A	N/A
	HBTEC-ALI	3/3	2/3	1/3	N/A	N/A
	Copies	5.7	5.7	7.5	N/A	N/A
HCoVHKU1	Days	7 days	53 days	74–116 days	137–158 days	202 days
	HNEpC-ALI	1/3	1/3	0/3	0/1	0/1
	Copies	8.7	8.7	N/A	N/A	N/A
	HBTEC-ALI	3/3	3/3	1/3	0/1	N/A
	Copies	8.8	8.5	7.6	N/A	N/A
RSV	Days	7 days	53–60 days	74–109 days	123–137 days	N/A
	HNEpC-ALI	0/6	3/6	1/6	1/2	N/A
	Copies	N/A	3.5	3.1	2.5	N/A
	HBTEC-ALI	4/6	1/6	0/4	1/1	N/A
	Copies	5.2	3.1	N/A	7.1	N/A
hMPV	Days	7 days	39–53 days	88–116 days	158 days	179–202 days
	HNEpC-ALI	0/8	0/8	0/7	0/2	0/2
	Copies	N/A	N/A	N/A	N/A	N/A
	HBTEC-ALI	1/6	0/6	0/5	1/2	N/A
	Copies	7.9	N/A	N/A	7.0	N/A
IAV	Days	7–11 days	18–53 days	67–102 days	N/A	N/A
	HNEpC-ALI	6/6	5/6	2/3	N/A	N/A
	Copies	7.0	7.6	6.1	N/A	N/A
	HBTEC-ALI	5/6	4/4	N/A	N/A	N/A
	Copies	7.3	8.2	N/A	N/A	N/A
IBV	Days	7–11 days	18–53 days	N/A	N/A	N/A
	HNEpC-ALI	4/6	6/6	N/A	N/A	N/A
	Copies	6.7	6.7	N/A	N/A	N/A
	HBTEC-ALI	5/6	2/4	N/A	N/A	N/A
	Copies	6.9	7.2	N/A	N/A	N/A
ICV	Days	7–11 days	53–60 days	74–109 days	123–158 days	N/A
	HNEpC-ALI	3/3	2/3	1/2	0/1	N/A
	Copies	8.2	7.7	7.1	N/A	N/A
	HBTEC-ALI	3/3	3/3	3/3	1/1	N/A
	Copies	8.0	7.7	7.9	8.8	N/A
HPIV1	Days	7 days	53–60 days	74–109 days	158 days	N/A
	HNEpC-ALI	1/3	2/3	1/2	0/1	N/A
	Copies	6.7	4.7	5.5	N/A	N/A
	HBTEC-ALI	3/3	3/3	2/2	N/A	N/A
	Copies	9.6	7.9	8.8	N/A	N/A
HPIV2	Days	7 days	53–60 days	74–109 days	123 days	N/A
	HNEpC-ALI	1/3	1/3	2/3	0/1	N/A
	Copies	8.1	8.7	7.6	N/A	N/A
	HBTEC-ALI	2/3	3/3	1/1	N/A	N/A
	Copies	7.2	7.0	8.5	N/A	N/A
HPIV3	Days	7 days	53–60 days	74–109 days	123 days	N/A
	HNEpC-ALI	3/3	3/3	2/2	0/1	N/A
	Copies	9.6	9.2	5.8	N/A	N/A
	HBTEC-ALI	3/3	3/3	2/2	N/A	N/A
	Copies	10.2	10.0	10.0	N/A	N/A
HPIV4	Days	7 days	53–60 days	74–109 days	123–158 days	N/A
	HNEpC-ALI	4/6	4/6	2/3	0/2	N/A
	Copies	6.9	6.0	7.1	N/A	N/A
	HBTEC-ALI	5/6	6/6	5/6	3/3	N/A
	Copies	7.1	7.3	7.1	7.5	N/A
ADVC	Days	7 days	18–60 days	67–74 days	N/A	N/A
	HNEpC-ALI	2/3	3/3	1/1	N/A	N/A
	Copies	10.1	10.2	10.5	N/A	N/A
	HBTEC-ALI	3/3	3/3	1/1	N/A	N/A
	Copies	9.9	10.2	10.6	N/A	N/A
ADVB3	Days	7 days	25–60 days	74–100 days	N/A	N/A
	HNEpC-ALI	3/3	3/3	3/3	N/A	N/A
	Copies	10.6	10.3	10.0	N/A	N/A
	HBTEC-ALI	3/3	3/3	N/A	N/A	N/A
	Copies	9.4	10.1	N/A	N/A	N/A
HBoV1	Days	7 days	25–60 days	81 days	N/A	N/A
	HNEpC-ALI	3/3	3/3	0/1	N/A	N/A
	Copies	10.0	10.1	N/A	N/A	N/A
	HBTEC-ALI	3/3	3/3	N/A	N/A	N/A
	Copies	10.1	9.5	N/A	N/A	N/A
HRVA	Days	7 days	53 days	109–116 days	137–158 days	N/A
	HNEpC-ALI	3/3	1/3	0/3	N/A	N/A
	Copies	9.1	7.0	N/A	N/A	N/A
	HBTEC-ALI	2/3	3/3	0/3	1/3	N/A
	Copies	7.9	5.1	N/A	4.6	N/A
HRVC	Days	7 days	53 days	74–116 days	123–158 days	N/A
	HNEpC-ALI	3/3	2/3	0/3	N/A	N/A
	Copies	8.8	8.1	N/A	N/A	N/A
	HBTEC-ALI	3/3	2/3	1/3	1/3	N/A
	Copies	8.5	7.9	6.9	7.4	N/A

^*a*^ Number of re-infection positive cases/total number tested.

^*b*^ N/A, Not applicable.

^*c*^ Average copy numbers (log copies per well) of replication-positive wells in re-infection experiment.

### Type I interferon secretion during long-term replication of respiratory viruses

**Fig 3:**
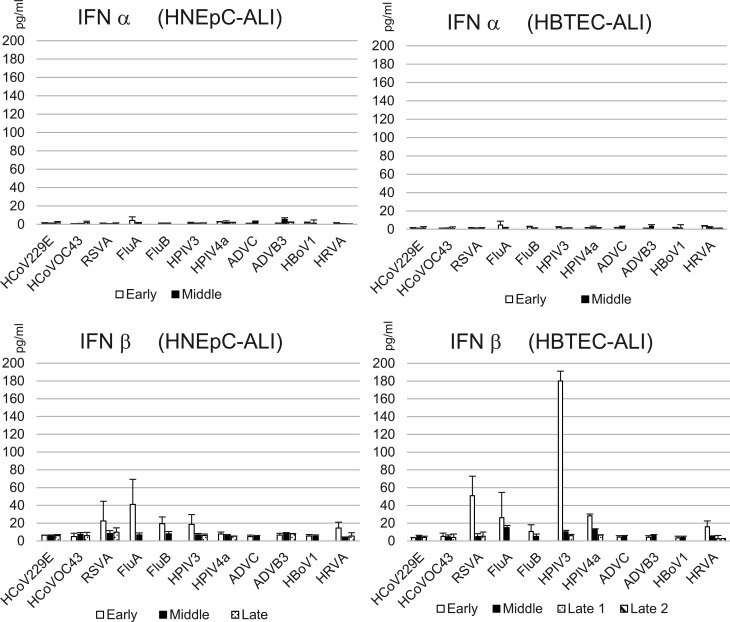
Expression of type I interferons. Levels of interferon-α and -β during long-term viral replication were measured using a commercial ELISA kit. Samples used in the re-infection experiments were analyzed, and data are presented as mean concentrations (pg/mL, *n* = 3). White bars represent samples from the early phase; black bars, the middle phase; and polka-dotted bars, the late phase.

As described above, respiratory viruses exhibited long-term replication in ALI cultures without being eliminated by host cell immunity. Type I interferons (IFNs) are key regulators of innate immune responses during viral infections. To assess IFN responses during long-term replication, secretion levels were measured by enzyme-linked immunosorbent assay (ELISA) using representative samples from the virus re-infection experiments (Fig. 3). IFNα secretion remained near the detection limit for all respiratory viruses tested. In contrast, IFNβ secretion was transiently elevated in the early phase of infection with RSV, Flu, HPIV3, and HRV in both ALI cultures. Among these, HPIV3 induced the most prominent IFNβ response. However, IFNβ levels declined after early phase and remained near the detection limit throughout the later phases. Notably, infections with HCoV, ADV, and HBoV1 did not elicit apparent IFN responses, even during the early phase. These findings suggest that ALI cultures develop tolerance to virus infections during long-term replication of respiratory viruses.

### Genetic stability during long-term viral isolation

As described above, most common respiratory viruses can replicate in human airway cells for over 100 days while retaining infectivity. To investigate whether prolonged replication affects viral genome integrity, we assessed the genetic stability of viruses maintained in long-term culture. Because the viruses used for experimental infection originated from various cell sources, virus isolation was first performed directly from clinical specimens using HBTEC-ALI cultures to minimize the influence of culture adaptation and better mimic primary infection. Genetic stability was then evaluated during long-term propagation using next-generation sequencing (NGS). Viruses that demonstrated relatively prolonged replication—HCoVs (OC43, HKU1, and NL63), pneumoviruses (RSV and hMPV), HPIVs, and HRVs—were selected based on availability. A summary of the time course of genetic changes is provided in Table 2, with detailed mutation profiles and schematics available in Files S1 and S2. While some viruses showed no detectable insertions or mutations during extended culture, most exhibited an increase in genetic variation after 50 to 60 days post-inoculation. Notably, RSV (OR371) and hMPV (OR642) accumulated a large number of mutations after 81 to 93 days. Most variations occurred in genes encoding surface structural proteins and polymerases, suggesting that repeated cycles of replication and reinfection may drive sequence diversification. These findings indicate that long-term replication of respiratory viruses, even in a controlled ALI culture environment, may increase the risk of genetic variation over time.

**TABLE 2:** Time course of genetic variations in long-cultured viruses during isolation in HBTEC-ALI cultures

Virus	Strain or subgroup	Isolate (accession)		Time course of variations				
				Early	Middle	Late 1	Late 2	Late 3
HCoV	OC43	O989	Days post-inoculation	**7** ^*a*^	28	56	84	N/A^*b*^
		(LC720429)	Numbers of variations	4	3	4	9	N/A
		OR427	Days post-inoculation	**7**	25	60	81	N/A
		(LC756670)	Numbers of variations	0	0	0	0	N/A
	HKU1	OR842	Days post-inoculation	**7**	32	60	95	N/A
		(LC817379)	Numbers of variations	0	0	0	0	N/A
	NL63	H257	Days post-inoculation	**4**	25	53	N/A	N/A
		(LC687394)	Numbers of variations	0	0	1	N/A	N/A
		O650	Days post-inoculation	**7**	28	56	84	N/A
		(LC720428)	Numbers of variations	0	0	1	3	N/A
RSV	B	HR128	Days post-inoculation	**11**	25	60	N/A	N/A
		(LC769227)	Numbers of variations	0	0	0	N/A	N/A
		OR371	Days post-inoculation	**7**	32	60	81	N/A
		(LC699634)	Numbers of variations	0	0	0	100	N/A
		OR809	Days post-inoculation	**7**	25	53	81	N/A
		(LC817403)	Numbers of variations	0	0	1	1	N/A
hMPV	B1	O53	Days post-inoculation	**18**	32	60	N/A	N/A
		(LC769210)	Numbers of variations	0	0	0	N/A	N/A
		OR677	Days post-inoculation	**32**	60	93	N/A	N/A
		(LC769218)	Numbers of variations	0	0	2	N/A	N/A
	B2	OR642	Days post-inoculation	**25**	60	93	123	N/A
		(LC769217)	Numbers of variations	0	0	18	20	N/A
HPIV	1	OR692	Days post-inoculation	**11**	25	53	81	N/A
		(LC817389)	Numbers of variations	0	0	1	1	N/A
		OR697	Days post-inoculation	**4**	25	60	93	123
		(LC769222)	Numbers of variations	0	0	1	1	1
		OR710	Days post-inoculation	**7**	25	53	81	N/A
		(LC817390)	Numbers of variations	0	0	0	0	N/A
	3	O716	Days post-inoculation	**11**	25	53	74	N/A
		(LC769224)	Numbers of variations	0	0	1	1	N/A
		OR381	Days post-inoculation	**7**	32	60	N/A	N/A
		(LC720880)	Numbers of variations	0	0	0	N/A	N/A
		OR913	Days post-inoculation	**7**	32	60	N/A	N/A
		(LC817398)	Numbers of variations	0	0	0	N/A	N/A
	4a	OH13	Days post-inoculation	**7**	25	53	81	N/A
		(LC817401)	Numbers of variations	0	0	0	0	N/A
	4b	OR476	Days post-inoculation	**7**	25	60	N/A	N/A
		(LC706556)	Numbers of variations	2	0	2	N/A	N/A
		OR487	Days post-inoculation	**7**	25	60	88	N/A
		(LC706557)	Numbers of variations	0	0	2	2	N/A
HBoV1		OR327	Days post-inoculation	**7**	32	60	N/A	N/A
		(LC720420)	Numbers of variations	0	1	2	N/A	N/A
		OR634	Days post-inoculation	**4**	11	32	N/A	N/A
		(LC769220)	Numbers of variations	0	0	0	N/A	N/A
HRV	A	O714//A81	Days post-inoculation	**11**	25	53	74	N/A
		(LC769225)	Numbers of variations	0	0	0	1	N/A
	C	OR463//C55	Days post-inoculation	**7**	25	60	96	N/A
		(LC720415)	Numbers of variations	0	0	3	3	N/A

^*a*^ The day on which the sequence was registered on GenBank is shown in bold.

^*b*^ N/A, Not applicable.

## DISCUSSION

This study demonstrates that most common respiratory viruses, excluding IAV, IBV, and DNA viruses, can replicate for approximately 100 days in ALI cultures of human airway epithelial cells while continuing to produce infectious progeny. Some viruses exhibited even longer replication periods, ranging from 150 to 200 days in culture. These findings suggest that many respiratory viruses have the potential to replicate and generate progeny viruses in airway tissues over extended periods—potentially lasting several months—if not actively eliminated by acquired immune responses. Notably, this prolonged replication capacity appears to be a shared feature across diverse viral families and species, with the exception of IAV, IBV, and DNA viruses. The turnover rate of respiratory epithelial cells is a key factor in determining the duration of viral persistence. Prior studies have reported that epithelial turnover in the lungs occurs approximately every 30–50 days (17, 18), while others have suggested a slower rate of 1 to 4 months (19). In ALI cultures, this slow turnover contributes to a highly stable epithelial system (20). Most respiratory viruses replicate in ALI cultures without inducing CPEs, supporting the possibility of continuous replication under stable tissue conditions. Considering the physiological turnover cycle of epithelial cells, these observations suggest that respiratory viruses may remain in the host for prolonged periods, provided they are not cleared by the immune system. In contrast, IAV, IBV and DNA viruses exhibited a shorter replication time than others. In contrast, IAV, IBV, and DNA viruses exhibited shorter replication durations. Previous studies have shown that IAV and IBV infections induce epithelial cell death via apoptosis and necrosis (21–23), while HBoV1 triggers pyroptosis (24) , and ADV induces lytic cell death to release progeny virions (25). These findings, together with our results, indicate that virus-induced cell death in these cases likely outpaces the natural turnover of epithelial cells, thereby limiting the duration of replication.

In a previous longitudinal cohort study involving repeated sampling and detection of respiratory viruses (11), median duration of viral detection was 1–2 weeks. However, HBoV1, HRV, and ADV were detectable for 14 to 39 weeks, while HCoVs, pneumoviruses, and respiroviruses were detectable for up to 4 to 6 weeks. These findings provide clinical evidence that extended respiratory virus detection can occur in healthy pediatric populations, even though respiratory viruses are typically cleared by the host immune system within approximately 2 weeks. In this study, type I interferons—key regulators of innate immunity in host cells—were not prominently induced during long-term infections, except for transient IFNβ secretion in the early phase, depending on the virus. These results suggest that ALI cultures, which lack effector immune cells, do not actively eliminate viruses through innate immune responses during long-term replication. The observation of only transient IFNβ responses supports the possibility of prolonged virus shedding in respiratory virus infections, particularly in immunocompromised individuals. This also highlights the importance of acquired immunity in effective clearance of respiratory viruses.

The long-term incubation and re-infection experiments conducted in this study revealed tissue-specific replication preferences and suggested a tendency toward latent or prolonged infection for certain viruses. Some clinical isolates, particularly those propagated in HBTEC-ALI cultures, did not induce CPEs in either ALI or conventional monolayer cultures, rendering CPE-based titration unreliable. Although influenza viruses and HBoV1 may eventually induce CPEs in ALI cultures, this typically requires 1–2 months, as shown in this study, making it impractical for routine titration. Focus assays using fluorescent antibody staining are also constrained by the need for virus-specific antisera and fixed time points, limiting standardization across virus types. Furthermore, the preparation of ALI cultures is time-intensive and cost-prohibitive, making large-scale virus titration studies unfeasible. For these reasons, we adopted time-course monitoring of viral genome replication by real-time RT-PCR as a standardized and practical method to assess replication dynamics across all clinical isolates.

In our model, influenza viruses exhibited shorter replication durations in HBTEC-ALI compared to HNEpC-ALI. It is well established that influenza virus infection can cause acute lung injury largely through robust immune responses (26, 27) and also induces epithelial cell apoptosis and necrosis (21). As ALI cultures lack immune cells, the observed cell death reflects direct virus-epithelium interactions. These results suggest that lung epithelial cells may be more vulnerable to influenza virus-induced cytotoxicity than nasal epithelial cells. HRVs demonstrated longer replication in HBTEC-ALI but higher re-infection rates in HNEpC-ALI. These findings are consistent with previous reports showing that HRVs are commonly detected in the upper respiratory tract (28) and associated with lower respiratory tract infections and asthma exacerbations (29, 30). Our results further align with studies reporting prolonged HRV infection in immunocompromised individuals (31, 32). Persistent adenovirus infection in the tonsillar tissue is known to contribute to adenoid hypertrophy (33, 34). In this study, ADVB3 showed longer replication in HNEpC-ALI than in HBTEC-ALI, suggesting its potential for prolonged infection not only in the lymphoid tissues but also in the nasal epithelial cells.

We previously reported that the occurrence of genetic mutations was significantly lower in ALI cultures than in conventional monolayer cell cultures during serial passage of HPIVs (35). However, the present study demonstrates that infections lasting more than 50 to 60 days can still lead to the accumulation of genetic mutations, even in the ALI culture system. Notably, recent reports have shown that long-term SARS-CoV-2 infections in immunocompromised patients are associated with the emergence of mutations in the spike (S) protein, contributing to the development of novel viral variants (36–38). Raglow et al. further observed that SARS-CoV-2 infections persisting beyond 56 days resulted in S protein mutations distinct from those observed in globally circulating strains (39). This 50–60-day threshold for the emergence of S protein mutations corresponds closely to the time frame during which spontaneous genetic changes began to accumulate in respiratory viruses in our ALI culture model. These findings suggest that even common respiratory viruses may undergo genetic evolution during extended replication in human airway tissues. Moreover, they raise the possibility that prolonged infections—particularly in immunocompromised individuals—could contribute to the emergence of novel viral variants. One possible explanation for the relatively low mutation frequency observed during early- and mid-phase replication in ALI cultures is the persistence of intact virions adhering to ciliated epithelial cells, potentially for several weeks to over 100 days following inoculation. This hypothesis warrants further investigation and highlights the need to study viral durability and retention in the mucosal environment of ALI cultures.

Collectively, our findings demonstrate that most common respiratory viruses possess the capacity to replicate continuously in human respiratory epithelial cells for extended periods, potentially on a monthly timescale. However, to reduce the risk of accumulating genetic mutations, transmission to new target cells should ideally occur within 50 to 60 days. These results provide novel insights into the potential for genetic evolution in common respiratory viruses during prolonged replication.

## MATERIALS AND METHODS

### Clinical specimens and viral detection

Clinical specimens were collected during the acute phase from pediatric inpatients with severe acute respiratory infections (SARIs) in Fukushima, Japan between 2018 and 2023 (5, 7). Nasopharyngeal swabs were obtained using a universal transport medium (Copan, Brescia, Italy) and stored at −80°C until analysis. No patient information other than disease classification was collected for this study. The study protocol was approved by the Ethics Committees of the National Institute of Infectious Diseases (approval numbers 816, 1001, 1087, and 1441) and Fukushima Medical University (approval number 29006). Verbal informed consent was obtained from participants or their guardians and documented in the medical records at each participating hospital. To ensure safe handling under biosafety level 2 (BSL-2) conditions, only SARS-CoV-2-negative specimens were used to evaluate common respiratory virus infections.

The total nucleic acids were extracted from specimens using the QIAamp 96 Virus QIAcube HT Kit (Qiagen, Hilden, Germany) according to the manufacturer’s instructions, except that the elution step was performed via centrifugation. Respiratory viruses were detected by real-time PCR or reverse transcription PCR (RT-PCR) using LightCycler instruments (Roche, Basel, Switzerland), as described previously (5, 14). Primer and probe sequences are provided in File S3. The following 17 viruses were tested: human orthopneumovirus (RSV subgroups A and B); IAV, IBV, and ICV; HCoV (229E, OC43, NL63, and HKU1); hMPV; HPIV (1, 2, 3, and 4); ADV (2 [for A, C, D, and F] and 4 [B and E]); HBoV1; and HRV.

### ALI cultures

ALI cultures of human respiratory epithelial cells (HBTEC-ALI and HNEpC-ALI) were established as described previously (12–14, 40). Briefly, human bronchial/tracheal epithelial cells (HBTEC; FC-0035, Lifeline Cell Technology, Frederick, MD, USA) or primary human nasal epithelial cells (HNEpC; C-12620, PromoCell, Heidelberg, Germany) were cultured in a 1:1 mixture of PneumaCult-EX and PneumaCult-EX Plus media (STEMCELL Technologies, Vancouver, Canada) and seeded onto 6.5-mm-diameter Transwell inserts (3470, Corning, One Riverfront Plaza, NY, USA). On the following day, the apical medium was removed, and the basal medium was replaced with a differentiation medium (PneumaCult-ALI, STEMCELL Technologies). The cultures were maintained under ALI conditions for 4 weeks, with weekly medium changes, to allow the formation of well-differentiated and polarized airway epithelial layers. All ALI cultures were incubated at 37°C in a humidified atmosphere containing 5% CO₂.

### Viruses

For infectious experiments, clinical isolates of the following respiratory viruses were used, all initially isolated using HBTEC-ALI cultures, unless otherwise indicated: HCoV-229E, Fukushima/H829/2020 (GenBank accession No. LC654445); HCoV-OC43, Tokyo/SGH-36/2014 (LC315646) (40); HCoV-NL63, Fukushima/OR236/2021 (LC687402); HCoV-HKU1, Tokyo/SGH-15/2014 (LC315650) (40); IAV(pdm), A/Fukushima/H469/2018 (H1N1) (LC720181–LC720188); IAV(H3), A/Fukushima/O571/2019 (H3N2) (LC720197–LC720204); IBV (Victoria), B/Fukushima/O654/2019 (LC720277–LC720284); IBV (Yamagata), B/Fukushima/H169/2018 (LC720269–LC720276); ICV, C/Fukushima/O181/2018 (LC720285–LC720291); HPIV1, PIV1/Fukushima/O81/2018 (LC720862) ; HPIV2, PIV2/Fukushima/O33/2018 (LC720865) (35); HPIV3, PIV3/Fukushima/O644/2019 (LC720877) (35); HPIV4a, PIV4a/Fukushima/O755/2019 (LC706551) (13); HPIV4b, PIV4b/Fukushima/OR487/2022 (LC706557) (13); ADVC, Fukushima/O1018/2019 (LC720425); ADVB3, Fukushima/O733/2019 (LC864042); HBoV1, Fukushima/H565/2019 (LC651171) (12); HRVA, HRV/A16/Fukushima/H561/2019 (LC699418) (41); and HRVC, HRV/C3/Fukushima/H399/2018 (LC699425) (41).

### Viral propagation and isolation

Virus isolation and propagation in HBTEC-ALI cultures were performed as follows: a 20 µL aliquot of clinical specimen or virus stock diluted 1:1 in 1% fetal calf serum (FCS)-supplemented DMEM containing antibiotics (penicillin–streptomycin, gentamicin, and amphotericin B) was applied to the apical surface of the ALI cultures. Cultures were incubated overnight at 34°C, after which the apical surface was washed four times with 1% FCS-DMEM. Because viral replication may take time to initiate, the fourth wash was retained as the day 0 (baseline) control. To prevent mycoplasma contamination, MC-210 (KAC Co., Ltd., Hyogo, Japan), a quinolone antimicrobial agent was continuously added to the basolateral medium throughout the incubation period, with weekly medium changes. Cells were subsequently washed on days 4, 7, 11, and every 7 days thereafter. Each apical wash was performed four times using 1% FCS-DMEM, and the collected washes were stored at −80°C. Viral replication was assessed by real-time RT-PCR based on crossing point (Cp) values, as previously described (5, 14). A sample was considered positive for virus replication if it showed an increase of >3.3 in Cp value (approximately equivalent to a one log₁₀ increase) compared to day 0 and/or 4. Copy numbers were calculated using standard curves generated from control templates prepared in a previous study (14). Results are presented as log₁₀ copies per well.

For pneumoviruses, RSV clinical isolates (RSV/A/NIID/2370/14 [LC474558] and RSV/B/NIID/2472/14 [LC474559]) (42) were obtained using HEp-2 cells. hMPV clinical isolates (hMPV-A/Tokyo/SGH-23/2018 [LC671555]; hMPV-B/Tokyo/SGH-20/2017 [LC671554]; and hMPV-B/Tokyo/SGH-30/2018 [LC671557]) (9) were obtained using VeroE6/TMPRSS2 cells. The virus stocks used in this study were propagated in the same cell lines in which they were originally isolated. Viral genome concentrations were quantified using real-time RT-PCR. All infection experiments were conducted at 34°C in a humidified incubator with 5% CO₂.

### Infectious experiments on ALI cultures

To evaluate the infectivity of viruses after long-term propagation, infectious experiments were performed using ALI cultures. Virus stocks (20 µL) with an average Cp value of 19 (mean ± SD: 19.18 ± 3.0) were diluted 1:1 in 1% FCS-DMEM supplemented with antibiotics (penicillin-streptomycin, gentamicin, and amphotericin B) and inoculated onto the apical surface of ALI cultures. After 4, 7, 11, and every 7 days thereafter, the apical surfaces were washed four times with 1% FCS-DMEM, and the collected washes were stored at −80°C for analysis. Viral replication was assessed by real-time RT-PCR as previously described. Cultures were considered negative for replication if the epithelial structure collapsed or if no increase in the viral copy number was observed in three consecutive samples. To test the infectivity of long-term cultured viruses, cell washes collected at specific time points were used for re-inoculation onto fresh ALI cultures. Samples obtained from HBTEC-ALI cultures were re-inoculated onto new HBTEC-ALI cultures, while those from HNEpC-ALI cultures were used to infect HNEpC-ALI cultures. Due to the limited number of ALI cultures available, infectivity was evaluated using representative time points. The duration of replication was categorized into three phases: early (7–11 days), middle (18–60 days), and late (67–202 days). The late phase was further divided into up to three subphases depending on the viral strain. Cell washes were collected on days 7, 18, and 32 post-infection and stored at −80°C. A culture was considered replication-positive (i.e., infectious) if it showed an increase in Cp value of >3.3 (approximately equivalent to one log₁₀) relative to day 0 and/or day 7 within 32 days of inoculation.

### Measurement of type I interferons

Type I interferons produced during long-term replication were measured using the VeriKine-HS Human Interferon Alpha All Subtype TCM ELISA Kit (Cat. No. 41135-1) and the VeriKine-HS Human Interferon Beta TCM ELISA Kit (Cat. No. 41435-1) (PBL Assay Science, Piscataway, NJ, USA) according to the manufacturer’s instructions. Samples collected during the re-infection experiments were used for the analysis.

### Virus isolation for sequencing analysis for genetic stability

To evaluate the genetic stability of viruses during long-term replication, samples were collected from newly conducted virus isolation experiments using HBTEC-ALI cultures. This approach was employed to minimize culture-adaptation bias and better simulate primary infection in a new host. Due to specimen availability constraints, the following virus groups were selected based on their demonstrated capacity for long-term replication: human coronaviruses (HCoVs; OC43, HKU1, NL63), pneumoviruses (RSV and hMPV), human parainfluenzaviruses (HPIVs), and human rhinoviruses (HRVs). Viral genome sequences were analyzed from successful long-term cultures, including two HCoV-OC43 isolates, two HCoV-NL63 isolates, one HCoV-HKU1 isolate, three RSV isolates, three hMPV isolates, three isolates for each HPIV type (HPIV1, HPIV3, HPIV4), two HBoV1 isolates, and two HRV isolates. As with the re-infection experiments, representative samples were selected for sequencing. The replication period for each virus was categorized into three to five temporal phases—early, middle, late 1, late 2, and late 3—depending on the replication profile of each specimen. Viral sequences obtained from the early phase were deposited in GenBank under the following accession numbers: (HCoVOC43: O989 [LC720429], OR427 [LC756670]; HCoVHKU1: OR842 [LC817379]; HCoVNL63: H257 [LC687394], O650 [LC720428]; RSV: HR128 [LC769227], OR371 [LC699634], OR809 [LC817403]; hMPV: O53 [LC769210], OR677 [LC769218], OR642 [LC769217]; HPIV1: OR692 [LC817389], OR697 [LC769222], OR710 [LC817390]; HPIV3: O716 [LC769224], OR381 [LC720880], OR913 [LC817398]; HPIV4: OH13 [LC817401], OR476 [LC706556], OR487 [LC706557]; HBoV1: OR327 [LC720420], OR634 [LC769220]; and HRV: O714 [LC769225], OR463 (LC720415].

### Sequencing analysis

NGS libraries were prepared using the NEBNext Ultra II RNA Library Prep Kit for Illumina (New England Biolabs [NEB], Ipswich, MA, USA) in accordance with the manufacturer’s instructions. Indexed libraries were sequenced using a 2 × 150 bp paired-end format on the DNBSEQ-G400 platform at AZENTA/GENEWIZ (Chelmsford, MA, USA). Raw sequencing reads were trimmed and subsequently either *de novo* assembled or mapped to their corresponding reference genomes using the CLC Genomics Workbench (versions 21.0.4 to 24.0.1; Qiagen) with default parameters. Genetic variations were analyzed using the Basic Variant Detection tool in the CLC software, with default settings applied for read mapping and variant calling.

### Statistical analysis

Statistical analyses were conducted using SigmaPlot software (version 14.5; Systat Software, Inc., Palo Alto, CA, USA). A *P*-value of less than 0.05 was considered statistically significant.

## Data Availability

The NGS raw reads were deposited under BioProject number PRJDB20463.

## SUPPLEMENTAL MATERIAL

File S1 msphere.00422-25-s0001.pdf Variants detected during long-term culture.

File S2 msphere.00422-25-s0002.pdf Schematic images of genetic stability during long-term viral isolation.

File S3 msphere.00422-25-s0003.pdf Primer/probe sequences used for the detection of respiratory viruses.

ASM does not own the copyrights to Supplemental Material that may be linked to, or accessed through, an article. The authors have granted ASM a non-exclusive, world-wide license to publish the Supplemental Material files. Please contact the corresponding author directly for reuse.
