# Supplementary material for: Potential for prolonged replication of common acute respiratory viruses in air-liquid interface cultures of primary human airway cells
Source: mSphere. 2025 Aug 28;10(9):e00422-25. doi: 10.1128/msphere.00422-25 (PMC12482148; doi:10.1128/msphere.00422-25)
Supplement: File S1 — Variants detected during long-term culture. [file msphere.00422-25-s0001.pdf]

Supplemental file 1

List of Variants Detected During Long-Term Culture

		Ref		Chromosome	Region	CDS	Type	Reference	Allele	Reference allele	Length	Linkage	Zygosity	Count	Coverage	Frequency
OC43	O989	7	7	OC43_Fukushima_O989_2019	22840^22841	hemagglutinin-esterase protein	Insertion	-	TAAGCTTAAGAA	No	12		Homozygous	24	35	68.57142857
OC43	O989	7	7	OC43_Fukushima_O989_2019	23489	hemagglutinin-esterase protein	SNV	C	T	No	1		Heterozygous	50	109	45.87155963
OC43	O989	7	7	OC43_Fukushima_O989_2019	23489	hemagglutinin-esterase protein	SNV	C	C	Yes	1		Heterozygous	59	109	54.12844037
OC43	O989	7	7	OC43_Fukushima_O989_2019	23705^23706	spike protein	Insertion	-	CGCTTAAAG	No	9		Homozygous	11	11	100
OC43	O989	7	7	OC43_Fukushima_O989_2019	24468	spike protein	SNV	T	A	No	1		Heterozygous	33	84	39.28571429
OC43	O989	7	7	OC43_Fukushima_O989_2019	24468	spike protein	SNV	T	T	Yes	1		Heterozygous	51	84	60.71428571
OC43	O989	7	28	OC43_Fukushima_O989_2019	22840^22841	hemagglutinin-esterase protein	Insertion	-	TAAGCTTAAGAA	No	12		Homozygous	2853	3691	77.29612571
OC43	O989	7	28	OC43_Fukushima_O989_2019	23489	hemagglutinin-esterase protein	SNV	C	T	No	1		Heterozygous	6413	9879	64.91547728
OC43	O989	7	28	OC43_Fukushima_O989_2019	23489	hemagglutinin-esterase protein	SNV	C	C	Yes	1		Heterozygous	3459	9879	35.01366535
OC43	O989	7	28	OC43_Fukushima_O989_2019	23705^23706	spike protein	Insertion	-	CGCTTAAAG	No	9		Homozygous	1156	1167	99.05741217
OC43	O989	7	56	OC43_Fukushima_O989_2019	22840^22841	hemagglutinin-esterase protein	Insertion	-	TAAGCTTAAGAA	No	12		Homozygous	302	418	72.24880383
OC43	O989	7	56	OC43_Fukushima_O989_2019	23489	hemagglutinin-esterase protein	SNV	C	T	No	1		Heterozygous	875	1579	55.41481951
OC43	O989	7	56	OC43_Fukushima_O989_2019	23489	hemagglutinin-esterase protein	SNV	C	C	Yes	1		Heterozygous	703	1579	44.52184927
OC43	O989	7	56	OC43_Fukushima_O989_2019	23705^23706	spike protein	Insertion	-	CGCTTAAAG	No	9		Homozygous	145	148	97.97297297
OC43	O989	7	56	OC43_Fukushima_O989_2019	24468	spike protein	SNV	T	A	No	1		Heterozygous	606	1512	40.07936508
OC43	O989	7	56	OC43_Fukushima_O989_2019	24468	spike protein	SNV	T	T	Yes	1		Heterozygous	905	1512	59.85449735
OC43	O989	7	84	OC43_Fukushima_O989_2019	11066	orf 1ab	SNV	C	T	No	1		Heterozygous	45	110	40.90909091
OC43	O989	7	84	OC43_Fukushima_O989_2019	11066	orf 1ab	SNV	C	C	Yes	1		Heterozygous	65	110	59.09090909
OC43	O989	7	84	OC43_Fukushima_O989_2019	11401	orf 1ab	SNV	G	T	No	1		Heterozygous	41	68	60.29411765
OC43	O989	7	84	OC43_Fukushima_O989_2019	11401	orf 1ab	SNV	G	G	Yes	1		Heterozygous	27	68	39.70588235
OC43	O989	7	84	OC43_Fukushima_O989_2019	11978	orf 1ab	SNV	T	C	No	1		Homozygous	110	138	79.71014493
OC43	O989	7	84	OC43_Fukushima_O989_2019	22840^22841	hemagglutinin-esterase protein	Insertion	-	TAAGCTTAAGAA	No	12		Homozygous	33	39	84.61538462
OC43	O989	7	84	OC43_Fukushima_O989_2019	24431	spike protein	SNV	C	T	No	1		Homozygous	104	123	84.55284553
OC43	O989	7	84	OC43_Fukushima_O989_2019	24468	spike protein	SNV	T	A	No	1		Homozygous	127	135	94.07407407
OC43	O989	7	84	OC43_Fukushima_O989_2019	24571	spike protein	SNV	C	T	No	1		Heterozygous	91	167	54.49101796
OC43	O989	7	84	OC43_Fukushima_O989_2019	24571	spike protein	SNV	C	C	Yes	1		Heterozygous	76	167	45.50898204
OC43	O989	7	84	OC43_Fukushima_O989_2019	25542	spike protein	SNV	C	T	No	1		Homozygous	111	129	86.04651163
OC43	O989	7	84	OC43_Fukushima_O989_2019	26346..26348	spike protein	Deletion	GTA	-	No	3		Homozygous	84	122	68.85245902
OC43	OR427	7	7													
OC43	OR427	7	25													
OC43	OR427	7	60													
OC43	OR427	7	81													
HKU1	OR842	7	7													
HKU1	OR842	7	32													
HKU1	OR842	7	60													
HKU1	OR842	7	95													
NL63	H257	4	4													
NL63	H257	4	25													
NL63	H257	4	53	HCoVNL63_Fukushima_H257_2018	21463	spike protein	SNV	C	T	No	1		Heterozygous	694	1430	48.53146853
NL63	H257	4	53	HCoVNL63_Fukushima_H257_2018	21463	spike protein	SNV	C	C	Yes	1		Heterozygous	733	1430	51.25874126
NL63	O650	28	7													
NL63	O650	28	28													
NL63	O650	28	56	NL63_Fukushima_O650_2019	21375	spike protein	SNV	C	T	No	1		Homozygous	580	587	98.80749574
NL63	O650	28	84	NL63_Fukushima_O650_2019	21375	spike protein	SNV	C	T	No	1		Homozygous	1391	1391	100

NL63	O650	28	84	NL63_Fukushima_O650_2019	21544	spike protein	SNV	T	C	No	1		Heterozygous	623	1020	61.07843137
NL63	O650	28	84	NL63_Fukushima_O650_2019	21544	spike protein	SNV	T	T	Yes	1		Heterozygous	396	1020	38.82352941

Ref				Chromosome	Region	CDS	Type	Reference	Allele	Reference allele	Length	Linkage	Zygosity	Count	Coverage	Frequency
RSV	HR128	11	7													
RSV	HR128	11	25													
RSV	HR128	11	60													
RSV	OR371	7	7													
RSV	OR371	7	32													
RSV	OR371	7	60													
RSV	OR371	7	81	RSA_Fukushima_OR371_2021	284	non-structural protein 1	SNV	T	C	No	1		Heterozygous	6556	18697	35.06444884
RSV	OR371	7	81	RSA_Fukushima_OR371_2021	284	non-structural protein 1	SNV	T	T	Yes	1		Heterozygous	12039	18697	64.39000909
RSV	OR371	7	81	RSA_Fukushima_OR371_2021	587	non-coding region	SNV	A	G	No	1		Heterozygous	2654	7291	36.40104238
RSV	OR371	7	81	RSA_Fukushima_OR371_2021	587	non-coding region	SNV	A	A	Yes	1		Heterozygous	4620	7291	63.36579344
RSV	OR371	7	81	RSA_Fukushima_OR371_2021	1019	non-coding region	SNV	A	C	No	1		Heterozygous	4552	12879	35.34435903
RSV	OR371	7	81	RSA_Fukushima_OR371_2021	1019	non-coding region	SNV	A	A	Yes	1		Heterozygous	8289	12879	64.360587
RSV	OR371	7	81	RSA_Fukushima_OR371_2021	1500	nucleocapsid protein	SNV	A	G	No	1		Heterozygous	3414	9416	36.25743415
RSV	OR371	7	81	RSA_Fukushima_OR371_2021	1500	nucleocapsid protein	SNV	A	A	Yes	1		Heterozygous	5975	9416	63.45581988
RSV	OR371	7	81	RSA_Fukushima_OR371_2021	1547	nucleocapsid protein	SNV	A	G	No	1		Heterozygous	3002	8489	35.36341147
RSV	OR371	7	81	RSA_Fukushima_OR371_2021	1547	nucleocapsid protein	SNV	A	A	Yes	1		Heterozygous	5464	8489	64.36564966
RSV	OR371	7	81	RSA_Fukushima_OR371_2021	1676	nucleocapsid protein	SNV	C	T	No	1		Heterozygous	4421	12255	36.0750714
RSV	OR371	7	81	RSA_Fukushima_OR371_2021	1676	nucleocapsid protein	SNV	C	C	Yes	1		Heterozygous	7805	12255	63.68829049
RSV	OR371	7	81	RSA_Fukushima_OR371_2021	1697	nucleocapsid protein	SNV	C	T	No	1		Heterozygous	4687	12990	36.08160123
RSV	OR371	7	81	RSA_Fukushima_OR371_2021	1697	nucleocapsid protein	SNV	C	C	Yes	1		Heterozygous	8259	12990	63.57967667
RSV	OR371	7	81	RSA_Fukushima_OR371_2021	1805	nucleocapsid protein	SNV	A	T	No	1		Heterozygous	4588	12674	36.20009468
RSV	OR371	7	81	RSA_Fukushima_OR371_2021	1805	nucleocapsid protein	SNV	A	A	Yes	1		Heterozygous	8044	12674	63.46851823
RSV	OR371	7	81	RSA_Fukushima_OR371_2021	2073	nucleocapsid protein	SNV	T	C	No	1		Heterozygous	6047	16950	35.67551622
RSV	OR371	7	81	RSA_Fukushima_OR371_2021	2073	nucleocapsid protein	SNV	T	T	Yes	1		Heterozygous	10867	16950	64.1120944
RSV	OR371	7	81	RSA_Fukushima_OR371_2021	2327	non-coding region	SNV	A	G	No	1		Heterozygous	3489	8755	39.85151342
RSV	OR371	7	81	RSA_Fukushima_OR371_2021	2327	non-coding region	SNV	A	A	Yes	1		Heterozygous	5244	8755	59.8972016
RSV	OR371	7	81	RSA_Fukushima_OR371_2021	2430	phosphoprotein	SNV	C	T	No	1		Heterozygous	5172	14525	35.60757315
RSV	OR371	7	81	RSA_Fukushima_OR371_2021	2430	phosphoprotein	SNV	C	C	Yes	1		Heterozygous	9310	14525	64.09638554
RSV	OR371	7	81	RSA_Fukushima_OR371_2021	4486	small hydrophobic protein	SNV	C	A	No	1		Heterozygous	4328	10958	39.49625844
RSV	OR371	7	81	RSA_Fukushima_OR371_2021	4486	small hydrophobic protein	SNV	C	C	Yes	1		Heterozygous	6601	10958	60.23909473
RSV	OR371	7	81	RSA_Fukushima_OR371_2021	4536	non-coding region	SNV	A	G	No	1		Heterozygous	3982	9872	40.3363047
RSV	OR371	7	81	RSA_Fukushima_OR371_2021	4536	non-coding region	SNV	A	A	Yes	1		Heterozygous	5871	9872	59.47123177
RSV	OR371	7	81	RSA_Fukushima_OR371_2021	4538	non-coding region	SNV	A	G	No	1		Heterozygous	3973	9799	40.54495357
RSV	OR371	7	81	RSA_Fukushima_OR371_2021	4538	non-coding region	SNV	A	A	Yes	1		Heterozygous	5802	9799	59.21012348
RSV	OR371	7	81	RSA_Fukushima_OR371_2021	4623	non-coding region	SNV	T	C	No	1		Heterozygous	2549	6458	39.47042428
RSV	OR371	7	81	RSA_Fukushima_OR371_2021	4623	non-coding region	SNV	T	T	Yes	1		Heterozygous	3886	6458	60.17342831
RSV	OR371	7	81	RSA_Fukushima_OR371_2021	4626	non-coding region	SNV	C	T	No	1		Heterozygous	2543	6406	39.69715891
RSV	OR371	7	81	RSA_Fukushima_OR371_2021	4626	non-coding region	SNV	C	C	Yes	1		Heterozygous	3824	6406	59.69403684

RSV	OR371	7	81	RSA_Fukushima_OR371_2021	4930	attachment glycoprotein	SNV	T	C	No	1		Heterozygous	7459	20066	37.17233131
RSV	OR371	7	81	RSA_Fukushima_OR371_2021	4930	attachment glycoprotein	SNV	T	T	Yes	1		Heterozygous	12566	20066	62.62334297
RSV	OR371	7	81	RSA_Fukushima_OR371_2021	5637	attachment glycoprotein	SNV	C	A	No	1		Heterozygous	2733	6914	39.52849291
RSV	OR371	7	81	RSA_Fukushima_OR371_2021	5637	attachment glycoprotein	SNV	C	C	Yes	1		Heterozygous	4155	6914	60.09545849
RSV	OR371	7	81	RSA_Fukushima_OR371_2021	5674	non-coding region	SNV	A	G	No	1		Heterozygous	2562	6622	38.68921776
RSV	OR371	7	81	RSA_Fukushima_OR371_2021	5674	non-coding region	SNV	A	A	Yes	1		Heterozygous	4051	6622	61.17487164
RSV	OR371	7	81	RSA_Fukushima_OR371_2021	5677	non-coding region	SNV	G	A	No	1		Heterozygous	2567	6569	39.07748516
RSV	OR371	7	81	RSA_Fukushima_OR371_2021	5677	non-coding region	SNV	G	G	Yes	1		Heterozygous	3993	6569	60.78550769
RSV	OR371	7	81	RSA_Fukushima_OR371_2021	5744	fusion protein	SNV	C	T	No	1		Heterozygous	3632	9121	39.82019515
RSV	OR371	7	81	RSA_Fukushima_OR371_2021	5744	fusion protein	SNV	C	C	Yes	1		Heterozygous	5473	9121	60.00438548
RSV	OR371	7	81	RSA_Fukushima_OR371_2021	5759	fusion protein	SNV	T	C	No	1		Heterozygous	3443	8953	38.45638334
RSV	OR371	7	81	RSA_Fukushima_OR371_2021	5759	fusion protein	SNV	T	T	Yes	1		Heterozygous	5501	8953	61.4430917
RSV	OR371	7	81	RSA_Fukushima_OR371_2021	5790	fusion protein	SNV	C	T	No	1		Heterozygous	3737	10037	37.23224071
RSV	OR371	7	81	RSA_Fukushima_OR371_2021	5790	fusion protein	SNV	C	C	Yes	1		Heterozygous	6285	10037	62.61831224
RSV	OR371	7	81	RSA_Fukushima_OR371_2021	5792	fusion protein	SNV	T	C	No	1		Heterozygous	3724	10042	37.08424617
RSV	OR371	7	81	RSA_Fukushima_OR371_2021	5792	fusion protein	SNV	T	T	Yes	1		Heterozygous	6300	10042	62.73650667
RSV	OR371	7	81	RSA_Fukushima_OR371_2021	5804	fusion protein	SNV	A	G	No	1		Heterozygous	3867	10251	37.72314896
RSV	OR371	7	81	RSA_Fukushima_OR371_2021	5804	fusion protein	SNV	A	A	Yes	1		Heterozygous	6364	10251	62.08174812
RSV	OR371	7	81	RSA_Fukushima_OR371_2021	6083	fusion protein	SNV	T	C	No	1		Heterozygous	3916	10918	35.86737498
RSV	OR371	7	81	RSA_Fukushima_OR371_2021	6083	fusion protein	SNV	T	T	Yes	1		Heterozygous	6978	10918	63.91280454
RSV	OR371	7	81	RSA_Fukushima_OR371_2021	6560	fusion protein	SNV	C	T	No	1		Heterozygous	3371	9321	36.16564746
RSV	OR371	7	81	RSA_Fukushima_OR371_2021	6560	fusion protein	SNV	C	C	Yes	1		Heterozygous	5859	9321	62.85806244
RSV	OR371	7	81	RSA_Fukushima_OR371_2021	6866	fusion protein	SNV	C	T	No	1		Heterozygous	3465	9680	35.79545455
RSV	OR371	7	81	RSA_Fukushima_OR371_2021	6866	fusion protein	SNV	C	C	Yes	1		Heterozygous	6177	9680	63.81198347
RSV	OR371	7	81	RSA_Fukushima_OR371_2021	6872	fusion protein	SNV	T	C	No	1		Heterozygous	3372	9596	35.13964152
RSV	OR371	7	81	RSA_Fukushima_OR371_2021	6872	fusion protein	SNV	T	T	Yes	1		Heterozygous	6204	9596	64.65193831
RSV	OR371	7	81	RSA_Fukushima_OR371_2021	7037	fusion protein	SNV	C	T	No	1		Heterozygous	3615	10015	36.09585622
RSV	OR371	7	81	RSA_Fukushima_OR371_2021	7037	fusion protein	SNV	C	C	Yes	1		Heterozygous	6334	10015	63.2451323
RSV	OR371	7	81	RSA_Fukushima_OR371_2021	7590	non-coding region	SNV	A	G	No	1		Heterozygous	1711	4864	35.17680921
RSV	OR371	7	81	RSA_Fukushima_OR371_2021	7590	non-coding region	SNV	A	A	Yes	1		Heterozygous	3143	4864	64.61759868
RSV	OR371	7	81	RSA_Fukushima_OR371_2021	7628	non-coding region	SNV	C	T	No	1		Heterozygous	2116	5104	41.45768025
RSV	OR371	7	81	RSA_Fukushima_OR371_2021	7628	non-coding region	SNV	C	C	Yes	1		Heterozygous	2962	5104	58.03291536
RSV	OR371	7	81	RSA_Fukushima_OR371_2021	7633	non-coding region	SNV	T	C	No	1		Heterozygous	2104	5122	41.07770402
RSV	OR371	7	81	RSA_Fukushima_OR371_2021	7633	non-coding region	SNV	T	T	Yes	1		Heterozygous	2992	5122	58.41468176
RSV	OR371	7	81	RSA_Fukushima_OR371_2021	7726	m2-1 protein	SNV	G	A	No	1		Heterozygous	3747	10248	36.56323185
RSV	OR371	7	81	RSA_Fukushima_OR371_2021	7726	m2-1 protein	SNV	G	G	Yes	1		Heterozygous	6474	10248	63.17330211
RSV	OR371	7	81	RSA_Fukushima_OR371_2021	8648	RNA-directed RNA polymerase L	SNV	C	T	No	1		Heterozygous	2253	5856	38.47336066
RSV	OR371	7	81	RSA_Fukushima_OR371_2021	8648	RNA-directed RNA polymerase L	SNV	C	C	Yes	1		Heterozygous	3566	5856	60.89480874
RSV	OR371	7	81	RSA_Fukushima_OR371_2021	8669..8670	RNA-directed RNA polymerase L	MNV	TG	CA	No	2		Heterozygous	2052	5789	35.44653653
RSV	OR371	7	81	RSA_Fukushima_OR371_2021	8669..8670	RNA-directed RNA polymerase L	MNV	TG	TG	Yes	2		Heterozygous	3690	5789	63.74157886

RSV	OR371	7	81	RSA_Fukushima_OR371_2021	8744	RNA-directed RNA polymerase L	SNV	T	A	No	1		Heterozygous	1984	5259	37.72580338
RSV	OR371	7	81	RSA_Fukushima_OR371_2021	8744	RNA-directed RNA polymerase L	SNV	T	T	Yes	1		Heterozygous	3270	5259	62.17912151
RSV	OR371	7	81	RSA_Fukushima_OR371_2021	8831	RNA-directed RNA polymerase L	SNV	A	G	No	1		Heterozygous	1906	4719	40.38991312
RSV	OR371	7	81	RSA_Fukushima_OR371_2021	8831	RNA-directed RNA polymerase L	SNV	A	A	Yes	1		Heterozygous	2780	4719	58.91078618
RSV	OR371	7	81	RSA_Fukushima_OR371_2021	8846	RNA-directed RNA polymerase L	SNV	G	A	No	1		Heterozygous	1866	4703	39.67680204
RSV	OR371	7	81	RSA_Fukushima_OR371_2021	8846	RNA-directed RNA polymerase L	SNV	G	G	Yes	1		Heterozygous	2820	4703	59.96172656
RSV	OR371	7	81	RSA_Fukushima_OR371_2021	8858	RNA-directed RNA polymerase L	SNV	T	G	No	1		Heterozygous	1879	4775	39.35078534
RSV	OR371	7	81	RSA_Fukushima_OR371_2021	8858	RNA-directed RNA polymerase L	SNV	T	T	Yes	1		Heterozygous	2875	4775	60.20942408
RSV	OR371	7	81	RSA_Fukushima_OR371_2021	8867	RNA-directed RNA polymerase L	SNV	A	G	No	1		Heterozygous	2053	4986	41.17529081
RSV	OR371	7	81	RSA_Fukushima_OR371_2021	8867	RNA-directed RNA polymerase L	SNV	A	A	Yes	1		Heterozygous	2907	4986	58.3032491
RSV	OR371	7	81	RSA_Fukushima_OR371_2021	8879	RNA-directed RNA polymerase L	SNV	T	C	No	1		Heterozygous	2125	4987	42.61078805
RSV	OR371	7	81	RSA_Fukushima_OR371_2021	8879	RNA-directed RNA polymerase L	SNV	T	T	Yes	1		Heterozygous	2845	4987	57.04832565
RSV	OR371	7	81	RSA_Fukushima_OR371_2021	8909	RNA-directed RNA polymerase L	SNV	T	C	No	1		Heterozygous	2075	4907	42.28652945
RSV	OR371	7	81	RSA_Fukushima_OR371_2021	8909	RNA-directed RNA polymerase L	SNV	T	T	Yes	1		Heterozygous	2822	4907	57.50968005
RSV	OR371	7	81	RSA_Fukushima_OR371_2021	8932	RNA-directed RNA polymerase L	SNV	C	T	No	1		Heterozygous	2444	5760	42.43055556
RSV	OR371	7	81	RSA_Fukushima_OR371_2021	8932	RNA-directed RNA polymerase L	SNV	C	C	Yes	1		Heterozygous	3270	5760	56.77083333
RSV	OR371	7	81	RSA_Fukushima_OR371_2021	8995	RNA-directed RNA polymerase L	SNV	A	T	No	1		Heterozygous	2595	6252	41.50671785
RSV	OR371	7	81	RSA_Fukushima_OR371_2021	8995	RNA-directed RNA polymerase L	SNV	A	A	Yes	1		Heterozygous	3655	6252	58.46129239
RSV	OR371	7	81	RSA_Fukushima_OR371_2021	9059	RNA-directed RNA polymerase L	SNV	C	T	No	1		Heterozygous	2543	6599	38.53614184
RSV	OR371	7	81	RSA_Fukushima_OR371_2021	9059	RNA-directed RNA polymerase L	SNV	C	C	Yes	1		Heterozygous	3942	6599	59.73632369
RSV	OR371	7	81	RSA_Fukushima_OR371_2021	9076	RNA-directed RNA polymerase L	SNV	G	A	No	1		Heterozygous	2507	6395	39.20250195
RSV	OR371	7	81	RSA_Fukushima_OR371_2021	9076	RNA-directed RNA polymerase L	SNV	G	G	Yes	1		Heterozygous	3861	6395	60.3752932
RSV	OR371	7	81	RSA_Fukushima_OR371_2021	9093	RNA-directed RNA polymerase L	SNV	C	T	No	1		Heterozygous	2363	6129	38.55441344
RSV	OR371	7	81	RSA_Fukushima_OR371_2021	9093	RNA-directed RNA polymerase L	SNV	C	C	Yes	1		Heterozygous	3718	6129	60.66242454
RSV	OR371	7	81	RSA_Fukushima_OR371_2021	9179	RNA-directed RNA polymerase L	SNV	T	C	No	1		Heterozygous	2323	6122	37.94511598
RSV	OR371	7	81	RSA_Fukushima_OR371_2021	9179	RNA-directed RNA polymerase L	SNV	T	T	Yes	1		Heterozygous	3773	6122	61.63018621
RSV	OR371	7	81	RSA_Fukushima_OR371_2021	9208	RNA-directed RNA polymerase L	SNV	G	A	No	1		Heterozygous	2158	5755	37.49782798
RSV	OR371	7	81	RSA_Fukushima_OR371_2021	9208	RNA-directed RNA polymerase L	SNV	G	G	Yes	1		Heterozygous	3557	5755	61.80712424
RSV	OR371	7	81	RSA_Fukushima_OR371_2021	9272	RNA-directed RNA polymerase L	SNV	C	T	No	1		Heterozygous	2431	6238	38.97082398
RSV	OR371	7	81	RSA_Fukushima_OR371_2021	9272	RNA-directed RNA polymerase L	SNV	C	C	Yes	1		Heterozygous	3791	6238	60.77268355
RSV	OR371	7	81	RSA_Fukushima_OR371_2021	9452	RNA-directed RNA polymerase L	SNV	G	A	No	1		Heterozygous	2608	7188	36.28269338
RSV	OR371	7	81	RSA_Fukushima_OR371_2021	9452	RNA-directed RNA polymerase L	SNV	G	G	Yes	1		Heterozygous	4555	7188	63.36950473
RSV	OR371	7	81	RSA_Fukushima_OR371_2021	9560	RNA-directed RNA polymerase L	SNV	G	A	No	1		Heterozygous	2626	7482	35.0975675
RSV	OR371	7	81	RSA_Fukushima_OR371_2021	9560	RNA-directed RNA polymerase L	SNV	G	G	Yes	1		Heterozygous	4831	7482	64.56829725
RSV	OR371	7	81	RSA_Fukushima_OR371_2021	9671	RNA-directed RNA polymerase L	SNV	A	G	No	1		Heterozygous	2952	7972	37.02960361
RSV	OR371	7	81	RSA_Fukushima_OR371_2021	9671	RNA-directed RNA polymerase L	SNV	A	A	Yes	1		Heterozygous	4986	7972	62.54390366
RSV	OR371	7	81	RSA_Fukushima_OR371_2021	9854	RNA-directed RNA polymerase L	SNV	C	T	No	1		Heterozygous	2985	8143	36.65725163
RSV	OR371	7	81	RSA_Fukushima_OR371_2021	9854	RNA-directed RNA polymerase L	SNV	C	C	Yes	1		Heterozygous	5111	8143	62.76556552
RSV	OR371	7	81	RSA_Fukushima_OR371_2021	10093	RNA-directed RNA polymerase L	SNV	G	A	No	1		Heterozygous	2613	7324	35.67722556
RSV	OR371	7	81	RSA_Fukushima_OR371_2021	10093	RNA-directed RNA polymerase L	SNV	G	G	Yes	1		Heterozygous	4685	7324	63.96777717

RSV	OR371	7	81	RSA_Fukushima_OR371_2021	10112	RNA-directed RNA polymerase L	SNV	G	A	No	1		Heterozygous	2484	7035	35.30916844
RSV	OR371	7	81	RSA_Fukushima_OR371_2021	10112	RNA-directed RNA polymerase L	SNV	G	G	Yes	1		Heterozygous	4533	7035	64.43496802
RSV	OR371	7	81	RSA_Fukushima_OR371_2021	10130	RNA-directed RNA polymerase L	SNV	A	G	No	1		Heterozygous	2603	7392	35.21374459
RSV	OR371	7	81	RSA_Fukushima_OR371_2021	10130	RNA-directed RNA polymerase L	SNV	A	A	Yes	1		Heterozygous	4749	7392	64.24512987
RSV	OR371	7	81	RSA_Fukushima_OR371_2021	10244	RNA-directed RNA polymerase L	SNV	C	T	No	1		Heterozygous	2178	6134	35.50701011
RSV	OR371	7	81	RSA_Fukushima_OR371_2021	10244	RNA-directed RNA polymerase L	SNV	C	C	Yes	1		Heterozygous	3937	6134	64.18324095
RSV	OR371	7	81	RSA_Fukushima_OR371_2021	10610	RNA-directed RNA polymerase L	SNV	C	T	No	1		Heterozygous	2443	6775	36.05904059
RSV	OR371	7	81	RSA_Fukushima_OR371_2021	10610	RNA-directed RNA polymerase L	SNV	C	C	Yes	1		Heterozygous	4298	6775	63.43911439
RSV	OR371	7	81	RSA_Fukushima_OR371_2021	10859	RNA-directed RNA polymerase L	SNV	A	G	No	1		Heterozygous	2532	6803	37.21887403
RSV	OR371	7	81	RSA_Fukushima_OR371_2021	10859	RNA-directed RNA polymerase L	SNV	A	A	Yes	1		Heterozygous	4252	6803	62.50183742
RSV	OR371	7	81	RSA_Fukushima_OR371_2021	10958	RNA-directed RNA polymerase L	SNV	A	G	No	1		Heterozygous	2662	7009	37.97974033
RSV	OR371	7	81	RSA_Fukushima_OR371_2021	10958	RNA-directed RNA polymerase L	SNV	A	A	Yes	1		Heterozygous	4323	7009	61.67784277
RSV	OR371	7	81	RSA_Fukushima_OR371_2021	11024	RNA-directed RNA polymerase L	SNV	T	C	No	1		Heterozygous	2621	7117	36.82731488
RSV	OR371	7	81	RSA_Fukushima_OR371_2021	11024	RNA-directed RNA polymerase L	SNV	T	T	Yes	1		Heterozygous	4479	7117	62.93382043
RSV	OR371	7	81	RSA_Fukushima_OR371_2021	11153	RNA-directed RNA polymerase L	SNV	G	A	No	1		Heterozygous	2426	6346	38.22880555
RSV	OR371	7	81	RSA_Fukushima_OR371_2021	11153	RNA-directed RNA polymerase L	SNV	G	G	Yes	1		Heterozygous	3891	6346	61.31421368
RSV	OR371	7	81	RSA_Fukushima_OR371_2021	11397	RNA-directed RNA polymerase L	SNV	T	C	No	1		Heterozygous	2134	5642	37.82346686
RSV	OR371	7	81	RSA_Fukushima_OR371_2021	11397	RNA-directed RNA polymerase L	SNV	T	T	Yes	1		Heterozygous	3477	5642	61.62708259
RSV	OR371	7	81	RSA_Fukushima_OR371_2021	11441	RNA-directed RNA polymerase L	SNV	C	A	No	1		Heterozygous	2277	5781	39.3876492
RSV	OR371	7	81	RSA_Fukushima_OR371_2021	11441	RNA-directed RNA polymerase L	SNV	C	C	Yes	1		Heterozygous	3469	5781	60.00691922
RSV	OR371	7	81	RSA_Fukushima_OR371_2021	11490	RNA-directed RNA polymerase L	SNV	T	C	No	1		Heterozygous	2240	5971	37.51465416
RSV	OR371	7	81	RSA_Fukushima_OR371_2021	11490	RNA-directed RNA polymerase L	SNV	T	T	Yes	1		Heterozygous	3690	5971	61.79869369
RSV	OR371	7	81	RSA_Fukushima_OR371_2021	11681	RNA-directed RNA polymerase L	SNV	A	G	No	1		Heterozygous	2498	6170	40.48622366
RSV	OR371	7	81	RSA_Fukushima_OR371_2021	11681	RNA-directed RNA polymerase L	SNV	A	A	Yes	1		Heterozygous	3654	6170	59.22204214
RSV	OR371	7	81	RSA_Fukushima_OR371_2021	11723	RNA-directed RNA polymerase L	SNV	T	C	No	1		Heterozygous	2997	7189	41.68869106
RSV	OR371	7	81	RSA_Fukushima_OR371_2021	11723	RNA-directed RNA polymerase L	SNV	T	T	Yes	1		Heterozygous	4171	7189	58.01919599
RSV	OR371	7	81	RSA_Fukushima_OR371_2021	11924	RNA-directed RNA polymerase L	SNV	G	A	No	1		Heterozygous	2950	7569	38.97476549
RSV	OR371	7	81	RSA_Fukushima_OR371_2021	11924	RNA-directed RNA polymerase L	SNV	G	G	Yes	1		Heterozygous	4584	7569	60.56282204
RSV	OR371	7	81	RSA_Fukushima_OR371_2021	12032	RNA-directed RNA polymerase L	SNV	A	T	No	1		Heterozygous	2752	7116	38.67341203
RSV	OR371	7	81	RSA_Fukushima_OR371_2021	12032	RNA-directed RNA polymerase L	SNV	A	A	Yes	1		Heterozygous	4358	7116	61.24227094
RSV	OR371	7	81	RSA_Fukushima_OR371_2021	12054	RNA-directed RNA polymerase L	SNV	G	T	No	1		Heterozygous	2558	6898	37.08321253
RSV	OR371	7	81	RSA_Fukushima_OR371_2021	12054	RNA-directed RNA polymerase L	SNV	G	G	Yes	1		Heterozygous	4286	6898	62.13395187
RSV	OR371	7	81	RSA_Fukushima_OR371_2021	12409	RNA-directed RNA polymerase L	SNV	C	A	No	1		Heterozygous	2516	6856	36.69778296
RSV	OR371	7	81	RSA_Fukushima_OR371_2021	12409	RNA-directed RNA polymerase L	SNV	C	C	Yes	1		Heterozygous	4321	6856	63.02508751
RSV	OR371	7	81	RSA_Fukushima_OR371_2021	12608	RNA-directed RNA polymerase L	SNV	C	T	No	1		Heterozygous	2684	7303	36.75201972
RSV	OR371	7	81	RSA_Fukushima_OR371_2021	12608	RNA-directed RNA polymerase L	SNV	C	C	Yes	1		Heterozygous	4581	7303	62.72764617
RSV	OR371	7	81	RSA_Fukushima_OR371_2021	12797	RNA-directed RNA polymerase L	SNV	C	T	No	1		Heterozygous	2579	6744	38.24139976
RSV	OR371	7	81	RSA_Fukushima_OR371_2021	12797	RNA-directed RNA polymerase L	SNV	C	C	Yes	1		Heterozygous	4150	6744	61.53618031
RSV	OR371	7	81	RSA_Fukushima_OR371_2021	12821	RNA-directed RNA polymerase L	SNV	C	T	No	1		Heterozygous	2576	6702	38.43628768
RSV	OR371	7	81	RSA_Fukushima_OR371_2021	12821	RNA-directed RNA polymerase L	SNV	C	C	Yes	1		Heterozygous	4102	6702	61.20561027

RSV	OR371	7	81	RSA_Fukushima_OR371_2021	13049	RNA-directed RNA polymerase L	SNV	A	G	No	1		Heterozygous	2494	6675	37.36329588
RSV	OR371	7	81	RSA_Fukushima_OR371_2021	13049	RNA-directed RNA polymerase L	SNV	A	A	Yes	1		Heterozygous	4160	6675	62.32209738
RSV	OR371	7	81	RSA_Fukushima_OR371_2021	13055	RNA-directed RNA polymerase L	SNV	A	T	No	1		Heterozygous	2528	7158	35.31712769
RSV	OR371	7	81	RSA_Fukushima_OR371_2021	13055	RNA-directed RNA polymerase L	SNV	A	A	Yes	1		Heterozygous	4581	7158	63.99832355
RSV	OR371	7	81	RSA_Fukushima_OR371_2021	13139	RNA-directed RNA polymerase L	SNV	T	C	No	1		Heterozygous	2818	7516	37.49334753
RSV	OR371	7	81	RSA_Fukushima_OR371_2021	13139	RNA-directed RNA polymerase L	SNV	T	T	Yes	1		Heterozygous	4678	7516	62.24055349
RSV	OR371	7	81	RSA_Fukushima_OR371_2021	13184	RNA-directed RNA polymerase L	SNV	T	C	No	1		Heterozygous	2554	6891	37.06283558
RSV	OR371	7	81	RSA_Fukushima_OR371_2021	13184	RNA-directed RNA polymerase L	SNV	T	T	Yes	1		Heterozygous	4313	6891	62.58888405
RSV	OR371	7	81	RSA_Fukushima_OR371_2021	13382	RNA-directed RNA polymerase L	SNV	C	T	No	1		Heterozygous	2873	7721	37.21020593
RSV	OR371	7	81	RSA_Fukushima_OR371_2021	13382	RNA-directed RNA polymerase L	SNV	C	C	Yes	1		Heterozygous	4809	7721	62.28467815
RSV	OR371	7	81	RSA_Fukushima_OR371_2021	13598	RNA-directed RNA polymerase L	SNV	T	C	No	1		Heterozygous	2406	6616	36.36638452
RSV	OR371	7	81	RSA_Fukushima_OR371_2021	13598	RNA-directed RNA polymerase L	SNV	T	T	Yes	1		Heterozygous	4178	6616	63.14993954
RSV	OR371	7	81	RSA_Fukushima_OR371_2021	13617	RNA-directed RNA polymerase L	SNV	C	T	No	1		Heterozygous	2362	6386	36.98715941
RSV	OR371	7	81	RSA_Fukushima_OR371_2021	13617	RNA-directed RNA polymerase L	SNV	C	C	Yes	1		Heterozygous	3985	6386	62.40212966
RSV	OR371	7	81	RSA_Fukushima_OR371_2021	13735	RNA-directed RNA polymerase L	SNV	A	G	No	1		Heterozygous	2575	6558	39.26501982
RSV	OR371	7	81	RSA_Fukushima_OR371_2021	13735	RNA-directed RNA polymerase L	SNV	A	A	Yes	1		Heterozygous	3917	6558	59.72857579
RSV	OR371	7	81	RSA_Fukushima_OR371_2021	13740	RNA-directed RNA polymerase L	SNV	A	G	No	1		Heterozygous	2541	6537	38.87104176
RSV	OR371	7	81	RSA_Fukushima_OR371_2021	13740	RNA-directed RNA polymerase L	SNV	A	A	Yes	1		Heterozygous	3981	6537	60.89949518
RSV	OR371	7	81	RSA_Fukushima_OR371_2021	13748	RNA-directed RNA polymerase L	SNV	A	G	No	1		Heterozygous	2502	6425	38.94163424
RSV	OR371	7	81	RSA_Fukushima_OR371_2021	13748	RNA-directed RNA polymerase L	SNV	A	A	Yes	1		Heterozygous	3914	6425	60.91828794
RSV	OR371	7	81	RSA_Fukushima_OR371_2021	13753	RNA-directed RNA polymerase L	SNV	G	A	No	1		Heterozygous	2591	6698	38.68318901
RSV	OR371	7	81	RSA_Fukushima_OR371_2021	13753	RNA-directed RNA polymerase L	SNV	G	G	Yes	1		Heterozygous	4066	6698	60.70468797
RSV	OR371	7	81	RSA_Fukushima_OR371_2021	13758	RNA-directed RNA polymerase L	SNV	C	T	No	1		Heterozygous	2685	6975	38.49462366
RSV	OR371	7	81	RSA_Fukushima_OR371_2021	13758	RNA-directed RNA polymerase L	SNV	C	C	Yes	1		Heterozygous	4263	6975	61.11827957
RSV	OR371	7	81	RSA_Fukushima_OR371_2021	13777..13778	RNA-directed RNA polymerase L	MNV	AC	GT	No	2		Heterozygous	2774	7482	37.07564822
RSV	OR371	7	81	RSA_Fukushima_OR371_2021	13777..13778	RNA-directed RNA polymerase L	MNV	AC	AC	Yes	2		Heterozygous	4571	7482	61.09329056
RSV	OR371	7	81	RSA_Fukushima_OR371_2021	13784	RNA-directed RNA polymerase L	SNV	G	A	No	1		Heterozygous	2953	7605	38.82971729
RSV	OR371	7	81	RSA_Fukushima_OR371_2021	13784	RNA-directed RNA polymerase L	SNV	G	G	Yes	1		Heterozygous	4622	7605	60.77580539
RSV	OR371	7	81	RSA_Fukushima_OR371_2021	13823	RNA-directed RNA polymerase L	SNV	G	A	No	1		Heterozygous	2906	7307	39.77008348
RSV	OR371	7	81	RSA_Fukushima_OR371_2021	13823	RNA-directed RNA polymerase L	SNV	G	G	Yes	1		Heterozygous	4354	7307	59.58669769
RSV	OR371	7	81	RSA_Fukushima_OR371_2021	13829	RNA-directed RNA polymerase L	SNV	T	A	No	1		Heterozygous	2873	7221	39.78673314
RSV	OR371	7	81	RSA_Fukushima_OR371_2021	13829	RNA-directed RNA polymerase L	SNV	T	T	Yes	1		Heterozygous	4339	7221	60.08863038
RSV	OR371	7	81	RSA_Fukushima_OR371_2021	13837	RNA-directed RNA polymerase L	SNV	G	A	No	1		Heterozygous	2760	6971	39.59259791
RSV	OR371	7	81	RSA_Fukushima_OR371_2021	13837	RNA-directed RNA polymerase L	SNV	G	G	Yes	1		Heterozygous	4186	6971	60.04877349
RSV	OR371	7	81	RSA_Fukushima_OR371_2021	13879	RNA-directed RNA polymerase L	SNV	T	C	No	1		Heterozygous	2794	6646	42.04032501
RSV	OR371	7	81	RSA_Fukushima_OR371_2021	13879	RNA-directed RNA polymerase L	SNV	T	T	Yes	1		Heterozygous	3817	6646	57.43304243
RSV	OR371	7	81	RSA_Fukushima_OR371_2021	14249	RNA-directed RNA polymerase L	SNV	T	A	No	1		Heterozygous	3999	10118	39.52362127
RSV	OR371	7	81	RSA_Fukushima_OR371_2021	14249	RNA-directed RNA polymerase L	SNV	T	T	Yes	1		Heterozygous	6089	10118	60.17987745
RSV	OR371	7	81	RSA_Fukushima_OR371_2021	14381	RNA-directed RNA polymerase L	SNV	T	C	No	1		Heterozygous	2869	7611	37.69544081
RSV	OR371	7	81	RSA_Fukushima_OR371_2021	14381	RNA-directed RNA polymerase L	SNV	T	T	Yes	1		Heterozygous	4710	7611	61.8841151

RSV	OR371	7	81	RSA_Fukushima_OR371_2021	14441	RNA-directed RNA polymerase L	SNV	A	G	No	1		Heterozygous	2782	7254	38.35125448
RSV	OR371	7	81	RSA_Fukushima_OR371_2021	14441	RNA-directed RNA polymerase L	SNV	A	A	Yes	1		Heterozygous	4451	7254	61.35925007
RSV	OR371	7	81	RSA_Fukushima_OR371_2021	14552	RNA-directed RNA polymerase L	SNV	T	C	No	1		Heterozygous	2279	6118	37.25073553
RSV	OR371	7	81	RSA_Fukushima_OR371_2021	14552	RNA-directed RNA polymerase L	SNV	T	T	Yes	1		Heterozygous	3797	6118	62.06276561
RSV	OR371	7	81	RSA_Fukushima_OR371_2021	14880	RNA-directed RNA polymerase L	SNV	C	T	No	1		Heterozygous	2742	7663	35.78233068
RSV	OR371	7	81	RSA_Fukushima_OR371_2021	14880	RNA-directed RNA polymerase L	SNV	C	C	Yes	1		Heterozygous	4893	7663	63.85227718
RSV	OR371	7	81	RSA_Fukushima_OR371_2021	15197	non-coding region	SNV	C	T	No	1		Heterozygous	411	1162	35.37005164
RSV	OR371	7	81	RSA_Fukushima_OR371_2021	15197	non-coding region	SNV	C	C	Yes	1		Heterozygous	747	1162	64.28571429
RSV	OR809	7	7													
RSV	OR809	7	25													
RSV	OR809	7	53	RSV_B_Fukushima_OR809_2023	5365	attachment glycoprotein	Deletion	A	-	No	1		Heterozygous	905	1781	50.81414935
RSV	OR809	7	53	RSV_B_Fukushima_OR809_2023	5365	attachment glycoprotein	SNV	A	A	Yes	1		Heterozygous	873	1781	49.01740595
RSV	OR809	7	81	RSV_B_Fukushima_OR809_2023	5365	attachment glycoprotein	Deletion	A	-	No	1		Heterozygous	2416	4038	59.8315998
RSV	OR809	7	81	RSV_B_Fukushima_OR809_2023	5365	attachment glycoprotein	SNV	A	A	Yes	1		Heterozygous	1619	4038	40.09410599

Ref				Chromosome	Region	CDS	Type	Reference	Allele	Reference allele	Length	Linkage	Zygosity	Count	Coverage	Frequency
hMPV	O53	18	18													
hMPV	O53	18	32													
hMPV	O53	18	60													
hMPV	OR642	25	25													
hMPV	OR642	25	60													
hMPV	OR642	25	93	HMPV_OR642_2022_B2	5704	small hydrophobic protein	SNV	T	C	No	1		Heterozygous	95	205	46.34146341
hMPV	OR642	25	93	HMPV_OR642_2022_B2	5704	small hydrophobic protein	SNV	T	T	Yes	1		Heterozygous	109	205	53.17073171
hMPV	OR642	25	93	HMPV_OR642_2022_B2	5785	small hydrophobic protein	SNV	T	C	No	1		Heterozygous	82	172	47.6744186
hMPV	OR642	25	93	HMPV_OR642_2022_B2	5785	small hydrophobic protein	SNV	T	T	Yes	1		Heterozygous	90	172	52.3255814
hMPV	OR642	25	93	HMPV_OR642_2022_B2	5807	small hydrophobic protein	SNV	T	C	No	1		Heterozygous	83	173	47.97687861
hMPV	OR642	25	93	HMPV_OR642_2022_B2	5807	small hydrophobic protein	SNV	T	T	Yes	1		Heterozygous	90	173	52.02312139
hMPV	OR642	25	93	HMPV_OR642_2022_B2	5847	small hydrophobic protein	SNV	T	C	No	1		Heterozygous	105	203	51.72413793
hMPV	OR642	25	93	HMPV_OR642_2022_B2	5847	small hydrophobic protein	SNV	T	T	Yes	1		Heterozygous	98	203	48.27586207
hMPV	OR642	25	93	HMPV_OR642_2022_B2	5871..5872	small hydrophobic protein	MNV	TT	CC	No	2		Heterozygous	97	220	44.09090909
hMPV	OR642	25	93	HMPV_OR642_2022_B2	5871..5872	small hydrophobic protein	MNV	TT	TT	Yes	2		Heterozygous	118	220	53.63636364
hMPV	OR642	25	93	HMPV_OR642_2022_B2	5879	small hydrophobic protein	SNV	T	C	No	1		Heterozygous	99	219	45.20547945
hMPV	OR642	25	93	HMPV_OR642_2022_B2	5879	small hydrophobic protein	SNV	T	T	Yes	1		Heterozygous	120	219	54.79452055
hMPV	OR642	25	93	HMPV_OR642_2022_B2	5918	small hydrophobic protein	SNV	T	C	No	1		Heterozygous	112	235	47.65957447
hMPV	OR642	25	93	HMPV_OR642_2022_B2	5918	small hydrophobic protein	SNV	T	T	Yes	1		Heterozygous	123	235	52.34042553
hMPV	OR642	25	93	HMPV_OR642_2022_B2	5966	small hydrophobic protein	SNV	T	C	No	1		Heterozygous	118	231	51.08225108
hMPV	OR642	25	93	HMPV_OR642_2022_B2	5966	small hydrophobic protein	SNV	T	T	Yes	1		Heterozygous	113	231	48.91774892
hMPV	OR642	25	93	HMPV_OR642_2022_B2	6016	non-coding region	SNV	T	C	No	1		Heterozygous	114	218	52.29357798
hMPV	OR642	25	93	HMPV_OR642_2022_B2	6016	non-coding region	SNV	T	T	Yes	1		Heterozygous	104	218	47.70642202
hMPV	OR642	25	93	HMPV_OR642_2022_B2	6029	non-coding region	SNV	T	C	No	1		Heterozygous	104	213	48.82629108
hMPV	OR642	25	93	HMPV_OR642_2022_B2	6029	non-coding region	SNV	T	T	Yes	1		Heterozygous	108	213	50.70422535
hMPV	OR642	25	93	HMPV_OR642_2022_B2	6040..6042	non-coding region	MNV	TTT	CCC	No	3		Heterozygous	96	207	46.37681159
hMPV	OR642	25	93	HMPV_OR642_2022_B2	6040..6042	non-coding region	MNV	TTT	TTT	Yes	3		Heterozygous	110	207	53.14009662
hMPV	OR642	25	93	HMPV_OR642_2022_B2	6510	attachment glycoprotein	SNV	T	C	No	1		Heterozygous	339	874	38.78718535
hMPV	OR642	25	93	HMPV_OR642_2022_B2	6510	attachment glycoprotein	SNV	T	T	Yes	1		Heterozygous	532	874	60.86956522
hMPV	OR642	25	93	HMPV_OR642_2022_B2	6523	attachment glycoprotein	SNV	T	C	No	1		Heterozygous	330	858	38.46153846
hMPV	OR642	25	93	HMPV_OR642_2022_B2	6523	attachment glycoprotein	SNV	T	T	Yes	1		Heterozygous	528	858	61.53846154
hMPV	OR642	25	93	HMPV_OR642_2022_B2	6539	attachment glycoprotein	SNV	T	C	No	1		Heterozygous	307	845	36.33136095
hMPV	OR642	25	93	HMPV_OR642_2022_B2	6539	attachment glycoprotein	SNV	T	T	Yes	1		Heterozygous	538	845	63.66863905
hMPV	OR642	25	93	HMPV_OR642_2022_B2	6770	attachment glycoprotein	SNV	T	C	No	1		Heterozygous	263	632	41.61392405
hMPV	OR642	25	93	HMPV_OR642_2022_B2	6770	attachment glycoprotein	SNV	T	T	Yes	1		Heterozygous	369	632	58.38607595
hMPV	OR642	25	93	HMPV_OR642_2022_B2	6837	attachment glycoprotein	SNV	T	C	No	1		Heterozygous	151	379	39.84168865

hMPV	OR642	25	93	HMPV_OR642_2022_B2	6837	attachment glycoprotein	SNV	T	T	Yes	1		Heterozygous	228	379	60.15831135
hMPV	OR642	25	93	HMPV_OR642_2022_B2	6847	attachment glycoprotein	SNV	T	C	No	1		Heterozygous	166	376	44.14893617
hMPV	OR642	25	93	HMPV_OR642_2022_B2	6847	attachment glycoprotein	SNV	T	T	Yes	1		Heterozygous	209	376	55.58510638
hMPV	OR642	25	93	HMPV_OR642_2022_B2	6901	non-coding region	Deletion	A	-	No	1		Heterozygous	60	143	41.95804196
hMPV	OR642	25	93	HMPV_OR642_2022_B2	6901	non-coding region	SNV	A	A	Yes	1		Heterozygous	83	143	58.04195804
hMPV	OR642	25	123	HMPV_OR642_2022_B2	5632^5633	small hydrophobic protein	Insertion	-	A	No	1		Heterozygous	553	1399	39.52823445
hMPV	OR642	25	123	HMPV_OR642_2022_B2	5632^5633	small hydrophobic protein	Insertion	-	-	Yes	0		Heterozygous	846	1399	60.47176555
hMPV	OR642	25	123	HMPV_OR642_2022_B2	5704	small hydrophobic protein	SNV	T	C	No	1		Homozygous	1063	1129	94.15411869
hMPV	OR642	25	123	HMPV_OR642_2022_B2	5785	small hydrophobic protein	SNV	T	C	No	1		Homozygous	1114	1196	93.14381271
hMPV	OR642	25	123	HMPV_OR642_2022_B2	5807	small hydrophobic protein	SNV	T	C	No	1		Homozygous	1244	1333	93.32333083
hMPV	OR642	25	123	HMPV_OR642_2022_B2	5847	small hydrophobic protein	SNV	T	C	No	1		Homozygous	1305	1405	92.88256228
hMPV	OR642	25	123	HMPV_OR642_2022_B2	5871..5872	small hydrophobic protein	MNV	TT	CC	No	2		Homozygous	1220	1319	92.49431387
hMPV	OR642	25	123	HMPV_OR642_2022_B2	5879	small hydrophobic protein	SNV	T	C	No	1		Homozygous	1244	1340	92.8358209
hMPV	OR642	25	123	HMPV_OR642_2022_B2	5918	small hydrophobic protein	SNV	T	C	No	1		Homozygous	1440	1524	94.48818898
hMPV	OR642	25	123	HMPV_OR642_2022_B2	5966	small hydrophobic protein	SNV	T	C	No	1		Homozygous	1435	1533	93.60730594
hMPV	OR642	25	123	HMPV_OR642_2022_B2	6016	non-coding region	SNV	T	C	No	1		Homozygous	1436	1525	94.16393443
hMPV	OR642	25	123	HMPV_OR642_2022_B2	6029	non-coding region	SNV	T	C	No	1		Homozygous	1445	1538	93.95318596
hMPV	OR642	25	123	HMPV_OR642_2022_B2	6040..6042	non-coding region	MNV	TTT	CCC	No	3		Homozygous	1296	1403	92.37348539
hMPV	OR642	25	123	HMPV_OR642_2022_B2	6510	attachment glycoprotein	SNV	T	C	No	1		Homozygous	2944	3140	93.75796178
hMPV	OR642	25	123	HMPV_OR642_2022_B2	6523	attachment glycoprotein	SNV	T	C	No	1		Homozygous	2842	3018	94.16832339
hMPV	OR642	25	123	HMPV_OR642_2022_B2	6539	attachment glycoprotein	SNV	T	C	No	1		Homozygous	2804	2985	93.93634841
hMPV	OR642	25	123	HMPV_OR642_2022_B2	6770	attachment glycoprotein	SNV	T	C	No	1		Homozygous	2070	2196	94.26229508
hMPV	OR642	25	123	HMPV_OR642_2022_B2	6788	attachment glycoprotein	SNV	T	C	No	1		Homozygous	1825	2045	89.24205379
hMPV	OR642	25	123	HMPV_OR642_2022_B2	6837	attachment glycoprotein	SNV	T	C	No	1		Homozygous	1395	1478	94.38430311
hMPV	OR642	25	123	HMPV_OR642_2022_B2	6847	attachment glycoprotein	SNV	T	C	No	1		Homozygous	1384	1463	94.60013671
hMPV	OR642	25	123	HMPV_OR642_2022_B2	6901	non-coding region	Deletion	A	-	No	1		Homozygous	744	955	77.90575916
hMPV	OR677	32	32													
hMPV	OR677	32	60													
hMPV	OR677	32	93	HMPV_OR677_2022_B1	5272	matrix protein 2-2	SNV	G	T	No	1		Heterozygous	9	21	42.85714286
hMPV	OR677	32	93	HMPV_OR677_2022_B1	5272	matrix protein 2-2	SNV	G	G	Yes	1		Heterozygous	12	21	57.14285714
hMPV	OR677	32	93	HMPV_OR677_2022_B1	5344	matrix protein 2-2	SNV	A	T	No	1		Heterozygous	6	11	54.54545455
hMPV	OR677	32	93	HMPV_OR677_2022_B1	5344	matrix protein 2-2	SNV	A	A	Yes	1		Heterozygous	5	11	45.45454545

		Ref		Chromosome	Region	CDS	Type	Reference	Allele	Reference allele	Length	Linkage	Zygosity	Count	Coverage	Frequency
PIV1	OR692	11	11													
PIV1	OR692	11	25													
PIV1	OR692	11	53	PIV1_Fukushima_OR692_2022	7306	hemagglutinin-neuraminidase	SNV	G	A	No	1		Heterozygous	556	862	64.50116009
PIV1	OR692	11	53	PIV1_Fukushima_OR692_2022	7306	hemagglutinin-neuraminidase	SNV	G	G	Yes	1		Heterozygous	305	862	35.38283063
PIV1	OR692	11	81	PIV1_Fukushima_OR692_2022	7306	hemagglutinin-neuraminidase	SNV	G	A	No	1		Homozygous	303	416	72.83653846
PIV1	OR697	4	4													
PIV1	OR697	4	25													
PIV1	OR697	4	60	HPIV1_OR697_2022	8525	hemagglutinin-neuraminidase	SNV	G	T	No	1		Heterozygous	446	927	48.11218986
PIV1	OR697	4	60	HPIV1_OR697_2022	8525	hemagglutinin-neuraminidase	SNV	G	G	Yes	1		Heterozygous	481	927	51.88781014
PIV1	OR697	4	93	HPIV1_OR697_2022	8525	hemagglutinin-neuraminidase	SNV	G	T	No	1		Heterozygous	388	852	45.5399061
PIV1	OR697	4	93	HPIV1_OR697_2022	8525	hemagglutinin-neuraminidase	SNV	G	G	Yes	1		Heterozygous	464	852	54.4600939
PIV1	OR697	4	123	HPIV1_OR697_2022	8525	hemagglutinin-neuraminidase	SNV	G	T	No	1		Heterozygous	837	1727	48.46554719
PIV1	OR697	4	123	HPIV1_OR697_2022	8525	hemagglutinin-neuraminidase	SNV	G	G	Yes	1		Heterozygous	886	1727	51.30283729
PIV1	OR710	7	7													
PIV1	OR710	7	25													
PIV1	OR710	7	53													
PIV1	OR710	7	81													
PIV3	O716	11	11													
PIV3	O716	11	25													
PIV3	O716	11	53	HPIV3_O716_2019	10555	L polymerase protein	SNV	T	C	No	1		Heterozygous	539	908	59.36123348
PIV3	O716	11	53	HPIV3_O716_2019	10555	L polymerase protein	SNV	T	T	Yes	1		Heterozygous	368	908	40.52863436
PIV3	O716	11	74	HPIV3_O716_2019	10555	L polymerase protein	SNV	T	C	No	1		Heterozygous	682	1211	56.31709331
PIV3	O716	11	74	HPIV3_O716_2019	10555	L polymerase protein	SNV	T	T	Yes	1		Heterozygous	528	1211	43.60033031
PIV3	OR381	7	7													
PIV3	OR381	7	32													
PIV3	OR381	7	60													
PIV3	OR913	7	7													
PIV3	OR913	7	32													
PIV3	OR913	7	60													
PIV4a	OH13	7	7													
PIV4a	OH13	7	25													
PIV4a	OH13	7	53													
PIV4a	OH13	7	81													
PIV4b	OR476	7	7	PIV4b_Fukushima_OR476_2022	8561	hemagglutinin-neuraminidase	SNV	A	G	No	1		Heterozygous	437	1229	35.55736371
PIV4b	OR476	7	7	PIV4b_Fukushima_OR476_2022	8561	hemagglutinin-neuraminidase	SNV	A	A	Yes	1		Heterozygous	790	1229	64.27990236
PIV4b	OR476	7	7	PIV4b_Fukushima_OR476_2022	9119	hemagglutinin-neuraminidase	SNV	C	T	No	1		Heterozygous	653	1809	36.09729132

PIV4b	OR476	7	7	PIV4b_Fukushima_OR476_2022	9119	hemagglutinin-neuraminidase	SNV	C	C	Yes	1		Heterozygous	1152	1809	63.68159204
PIV4b	OR476	7	25													
PIV4b	OR476	7	60	PIV4b_Fukushima_OR476_2022	2419	phosphoprotein	SNV	A	T	No	1		Homozygous	16	22	72.72727273
PIV4b	OR476	7	60	PIV4b_Fukushima_OR476_2022	8152	hemagglutinin-neuraminidase	SNV	G	A	No	1		Homozygous	24	33	72.72727273
PIV4b	OR487	7	7													
PIV4b	OR487	7	25													
PIV4b	OR487	7	60	PIV4b_Fukushima_OR487_2022	3858	matrix protein	SNV	T	G	No	1		Heterozygous	1362	3786	35.97464342
PIV4b	OR487	7	60	PIV4b_Fukushima_OR487_2022	3858	matrix protein	SNV	T	T	Yes	1		Heterozygous	2423	3786	63.99894348
PIV4b	OR487	7	60	PIV4b_Fukushima_OR487_2022	8154	hemagglutinin-neuraminidase	SNV	G	A	No	1		Heterozygous	2045	3474	58.86586068
PIV4b	OR487	7	60	PIV4b_Fukushima_OR487_2022	8154	hemagglutinin-neuraminidase	SNV	G	G	Yes	1		Heterozygous	1429	3474	41.13413932
PIV4b	OR487	7	88	PIV4b_Fukushima_OR487_2022	3858	matrix protein	SNV	T	G	No	1		Heterozygous	588	1128	52.12765957
PIV4b	OR487	7	88	PIV4b_Fukushima_OR487_2022	3858	matrix protein	SNV	T	T	Yes	1		Heterozygous	540	1128	47.87234043
PIV4b	OR487	7	88	PIV4b_Fukushima_OR487_2022	8154	hemagglutinin-neuraminidase	SNV	G	A	No	1		Homozygous	906	1101	82.28882834

Ref				Chromosome	Region	CDS	Type	Reference	Allele	Reference allele	Length	Linkage	Zygosity	Count	Coverage	Frequency
HBoV1	OR327	7	7													
HBoV1	OR327	7	32	HBoV_Fukushima_OR327_2021	204	non-coding	SNV	A	G	No	1		Heterozygous	6	16	37.5
HBoV1	OR327	7	32	HBoV_Fukushima_OR327_2021	204	non-coding	SNV	A	A	Yes	1		Heterozygous	10	16	62.5
HBoV1	OR327	7	60	HBoV_Fukushima_OR327_2021	5390..5392	non-coding	MNV	AAA	TTT	No	3		Heterozygous	176	372	47.31182796
HBoV1	OR327	7	60	HBoV_Fukushima_OR327_2021	5390..5392	non-coding	MNV	AAA	AAA	Yes	3		Heterozygous	195	372	52.41935484
HBoV1	OR327	7	60	HBoV_Fukushima_OR327_2021	5411	non-coding	SNV	C	G	No	1		Homozygous	187	200	93.5
HBoV1	OR634	11	4													
HBoV1	OR634	11	11													
HBoV1	OR634	11	32													

		Ref		Chromosome	Region	CDS	Type	Reference	Allele	Reference allele	Length	Linkage	Zygosity	Count	Coverage	Frequency
HRV	O714	11	11													
HRV	O714	11	25													
HRV	O714	11	53													
HRV	O714	11	74	HRV_O714_2019_A81	4982	3A	SNV	G	T	No	1		Heterozygous	6	14	42.85714286
HRV	O714	11	74	HRV_O714_2019_A81	4982	3A	SNV	G	G	Yes	1		Heterozygous	8	14	57.14285714
HRV	OR463	7	7													
HRV	OR463	7	25													
HRV	OR463	7	60	RVC_Fukushima_OR463_2021_C55	2000	1C	SNV	G	A	No	1		Heterozygous	9	25	36
HRV	OR463	7	60	RVC_Fukushima_OR463_2021_C55	2000	1C	SNV	G	G	Yes	1		Heterozygous	16	25	64
HRV	OR463	7	60	RVC_Fukushima_OR463_2021_C55	2392	1D	SNV	C	T	No	1		Homozygous	30	33	90.90909091
HRV	OR463	7	60	RVC_Fukushima_OR463_2021_C55	5237	3C	SNV	G	A	No	1		Homozygous	31	33	93.93939394
HRV	OR463	7	96	RVC_Fukushima_OR463_2021_C55	2950	1D	SNV	C	T	No	1		Heterozygous	52	107	48.59813084
HRV	OR463	7	96	RVC_Fukushima_OR463_2021_C55	2950	1D	SNV	C	C	Yes	1		Heterozygous	55	107	51.40186916
HRV	OR463	7	96	RVC_Fukushima_OR463_2021_C55	3074	1D	SNV	T	C	No	1		Homozygous	102	117	87.17948718
HRV	OR463	7	96	RVC_Fukushima_OR463_2021_C55	6919	3D	SNV	C	T	No	1		Homozygous	122	145	84.13793103
