# Supplementary material for: Potential for prolonged replication of common acute respiratory viruses in air-liquid interface cultures of primary human airway cells
Source: mSphere. 2025 Aug 28;10(9):e00422-25. doi: 10.1128/msphere.00422-25 (PMC12482148; doi:10.1128/msphere.00422-25)

Supplemental file 2

Schematic images of genetic stability during long-term viral isolation

HCoV NL63 H257

Early(7d)

Middle(25-28d)

Late 1 (53-56d)

Late 2 (84d)

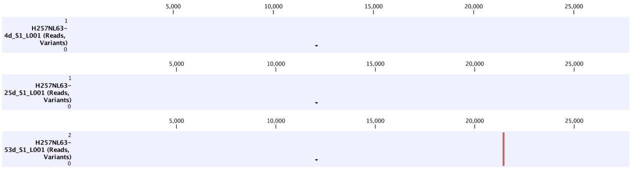

HCoV OC43 OR427

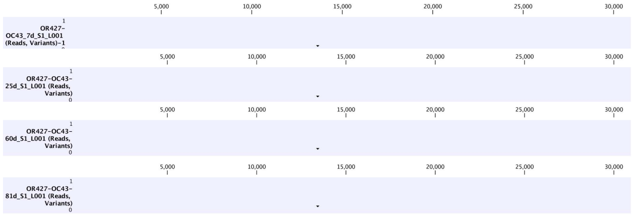

HCoV NL63 O650

Early(7d)

Middle(25-32d)

Late 1 (60d)

Late 2 (81-95d)

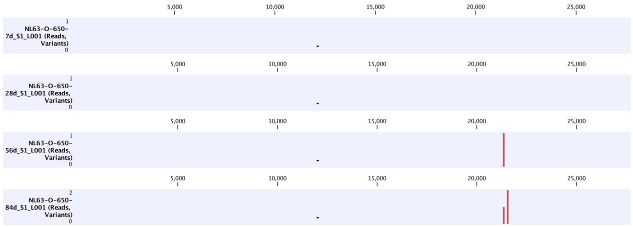

HCoV OC43 O989

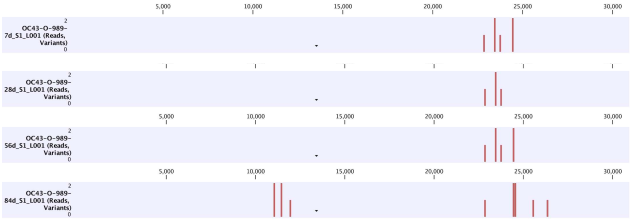

HCoV HKU1 OR842

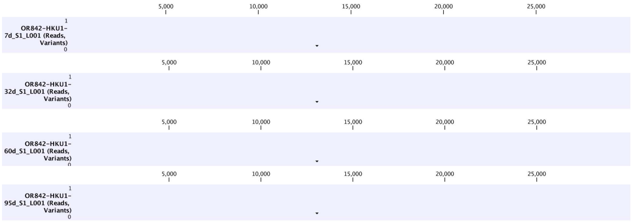

HBoV1 OR327

Early(7d)

Middle(32d)

Late 1 (60d)

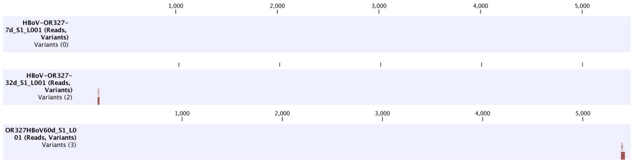

HBoV1 OR634

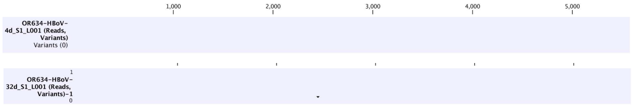

Schematic images of genetic stability during long-term viral isolation

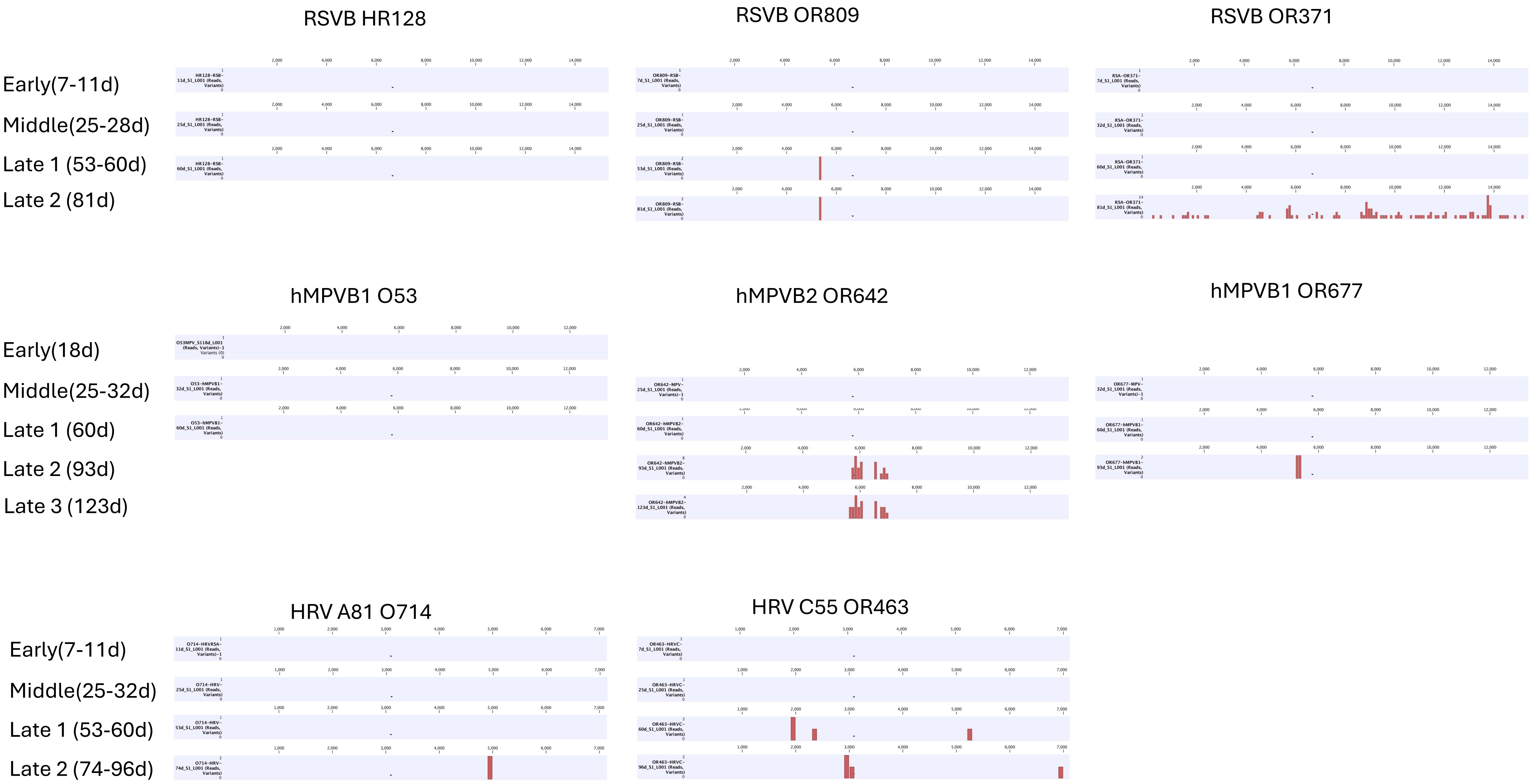

Schematic images of genetic stability during long-term viral isolation

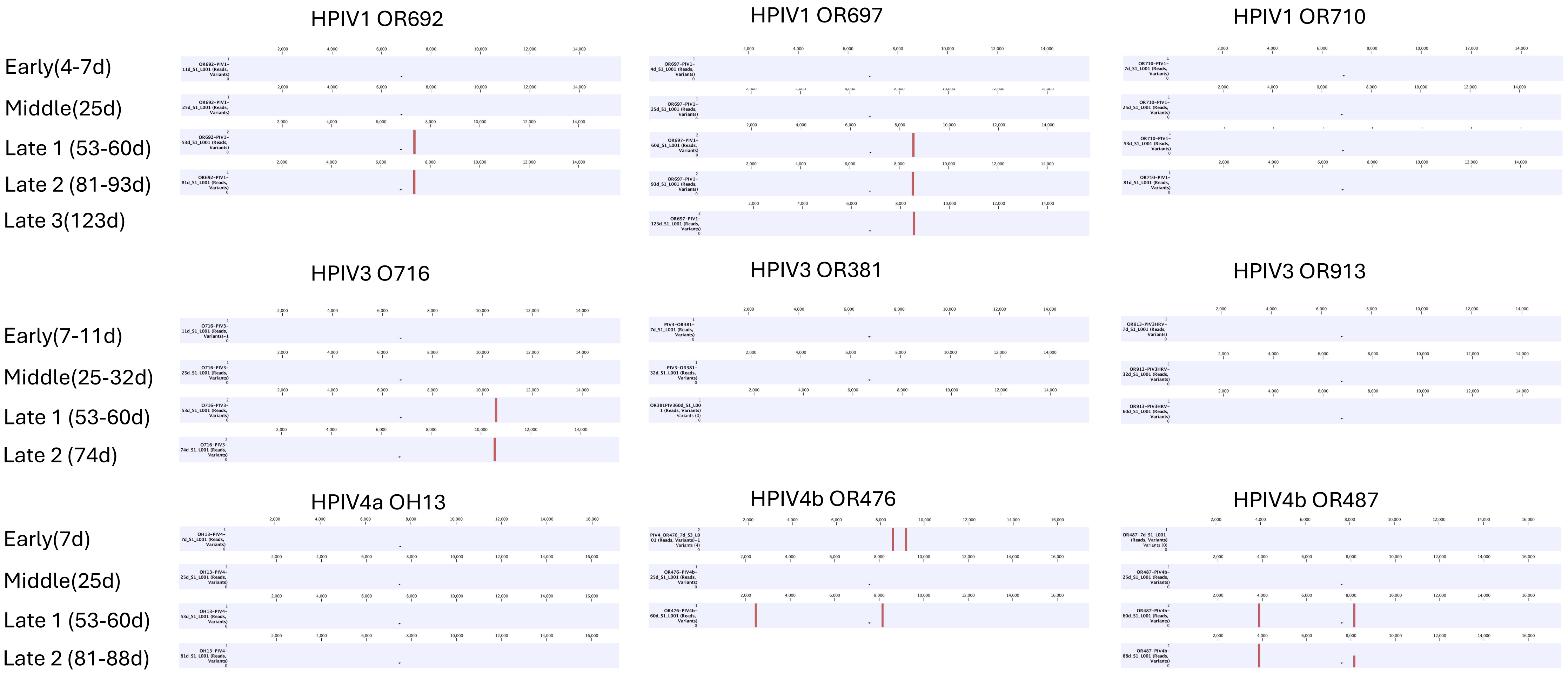

Supplement: File S2 — Schematic images of genetic stability during long-term viral isolation. [file msphere.00422-25-s0002.pdf]
