# Supplementary material for: Potential for prolonged replication of common acute respiratory viruses in air-liquid interface cultures of primary human airway cells
Source: mSphere. 2025 Aug 28;10(9):e00422-25. doi: 10.1128/msphere.00422-25 (PMC12482148; doi:10.1128/msphere.00422-25)
Supplement: File S3 — Primer/probe sequences used for the detection of respiratory viruses. [file msphere.00422-25-s0003.pdf]

Supplemental file 3. Primer/probe sequences used for the detection of respiratory viruses.

Target (modification)	Primer/Probe names	Sequence (5' to 3')	Target	Length	Reference
hMPV	hMPV F	ARYTGCCRATCTTTGGBGTYATAG	fusion	24	(1) Modified
	hMPV R	TYTKACAATACCAYCCTTGRTCYTC		25	
FAM-BHQ1	hMPV Probe	MAARGCAGCYCCYTCTTGYTCMGRA		25	
RSV	HRSV-F	ATGGCTCTTAGCAAAGTCAAGT	Matrix	22	(2), (3).
	HRSV-R	TGCACATCATAATTRGGAGTRTCA		24	
FAM-**BHQ1**-Phosphate	HRSV A-P	ACACTCAACAAAGA**T**CAACTTCTRTCATCCAGCA		34	
TEXsRED-**BHQ3**-Phosphate	HRSV B-P	ACATTAAATAAGGA**T**CAGCTGCTGTCATCCAGCA		34	
HBoV	HBoV 2693f	GTCAACACAGAGCTTCCAATCC	NP-1	22	(4)
	HBoV 2781r	TGAATTAGTACCATCTCTAGCAATGC		26	
FAM-BHQ1	HBoV 2724-TP	AGTGCCAGTAGAACCCACACCACCCT		26	
Rhino	Pic-1	TCCTCCGGCCCCTGAAT	5' NTR	17	(5)
	Pic-3	GAAACACGGACACCCAAAGTAGT		23	
FAM-MGB	Pic-5-Probe	YGGCTAACCYWAACCC		16	
HCoV (NL63)	HCoV-NL63_N-F	CTCTTTCTCAACCCAGGGCTG	Nucleocapsid	21	(6)
	HCoV-NL63_N-R	CGAGGACCAAAGCACTGAATAAC		23	
FAM-BHQ1	HCoV-NL63_N-Probe	ACCTCGTTGGAAGCGTGTTCCTACCA		26	
HCoV (OC43)	HCoV-OC43_N-F	GGGTACTGGTACAGACACAACAG	Nucleocapsid	23	(6)
	HCoV-OC43_N-R	GGTGCCGTACTGGTCTTTAGC		21	
Cy5-BHQ3	HCoV-	CCGATGGCAACCAGCGTCAACTGCT		25	

	OC43_N-Probe				
HCoV (HKU1)	HCoV-HKU1_N-F	GTTGCTAATCACCAAGCTGACAC	Nucleocapsid	23	(7)
	HCoV-HKU1_N-R	CGTACCAGGCGGAAACCTAG		20	
FAM-BHQ1	HCoV-HKU1_N-Probe	CCCTCCGATGTTTCGTCAAGGGATCCT		27	
HCoV (229E)	HCoV-229E-Foward	CAGTCAAATGGGCTGATGCA	Nucleoprotein	20	(8, 9)
	HCoV-229E-Reverse	AAAGGGCTATAAAGAGAATAAGGTATTCT		29	
VIC-TAMRA	HCoV-229E-Probe	CCCTGACGACCACGTTGTGGTTCA		24	
Adenovirus 2 (A, C, D and F))	Ad2-F	CCAGGACGCCTCGGAGTA	Hexon	18	(10)
	Ad2-R	AAACTTGTTATTCAGGCTGAAGTACGT		27	
FAM-BHQ1	Ad2-probe	AGTTTGCCCGCGCCACCG		18	
Adenovirus 4 (B and E)	Ad4-F	GGACAGGACGCTTCGGAGTA	Hexon	20	(10)
	Ad4-R	CTTGTTCCCCAGACTGAAGTAGGT		24	
Cy5-BHQ3	Ad4-probe	CAGTTCGCCCGYGCMACAG		19	
HPIV1	HPIV1_F	CCATCCTTTTTCTGCAATGTATCC	HN	24	(6)
	HPIV1_R	ATTGCAAACACTCTGATTAACATTGG		26	
Cy5-BHQ3	HPIV1_P	CGGTGGCTTAACAACTCCGCTCCAAGG		27	
HPIV2	HPIV2_F	GGACGCCTAAATATGGACCTCTC	HN	23	(6)
	HPIV2_R	GTGAGTGTAACACCAATGGGTCT		23	
Cy5-BHQ3	HPIV2_probe	CCCAGCTTTATCCCCTCAGCAACATCTCCC		30	
HPIV3	HPIV-3 F	ATGGACATGGCATAATGTGCTAT	HN	23	(6)
	HPIV-3 R	AATGCTYCCTGTGGGATTGAG		21	
FAM-BHQ1	HPIV-3 Probe	TCCCCATGGACATTCATTGTTTCCTGGTCT		30	
HPIV4	HPIV-4AB_F	CAAAYGATCCACAGCAAAGATTC	Nucleoprotein	23	(6) Modified
	HPIV-4AB_R	ATGTGGCCTGTAAGGAAAGCA		21	
VIC-MGB	HPIV-4AB_P	GTATCATCATCTGCCAAAT		19	
FluC	Flu C F	GACGACTACACACCAGACATCC	Matrix	22	(6)

	Flu C R	CTGAGACATTACTCCTGTATCTTTCAC		27	
Cy5-BHQ3	Flu C Probe	TTGCATCTCAACCAAGCTGTGATTGTTCCT		30	
FluA	MP-39-67For	CCMAGGTCGAAACGTAYGTTCTCTCTATC		29	
	MP-183-153Rev	TGACAGRATYGGTCTTGTCTTTAGCCAYTCCA	Matrix	32	(11)
FAM-MGB	MP-96-75ProbeAs	ATYTCGGCTTTGAGGGGGCCTG		22	
FluB	NIID-TypeB TMPrimer-F1	GGAGCAACCAATGCCAC		30	
	NIID-TypeB TMPrimer-R1	GTKTAGGCGGTCTTGACCAG	NS	22	(11)
FAM-MGB	NIID-TypeB Probe2	ATAAACTTYGAAGCAGGAAT		23	

Red character shows labeled fluorescences (5' to 3')

References

1. Kaida A, Kubo H, Shiomi M, Kohdera U, Iritani N. Evaluation of real-time RT-PCR compared with conventional RT-PCR for detecting human metapneumovirus RNA from clinical specimens. Japanese journal of infectious diseases. 2008 Nov;61(6):461-4.

2. Wang L, Piedra PA, Avadhanula V, Durigon EL, Machablishvili A, Lopez MR, et al. Duplex real-time RT-PCR assay for detection and subgroup-specific identification of human respiratory syncytial virus. J Virol Methods. 2019 Sep;271:113676.

3. Suwa R, Kume Y, Kawase M, Chishiki M, Ono T, Norito S, et al. Practical Validation of United States Centers for Disease Control and Prevention Assays for the Detection of Human Respiratory Syncytial Virus in Pediatric Inpatients in Japan. Pathogens. 2022;11(7).

4. Kaida A, Kubo H, Takakura K, Iritani N. Detection and quantitative analysis of human bocavirus associated with respiratory tract infection in Osaka City, Japan. Microbiol Immunol. 2010 May;54(5):276-81.

5. Do DH, Laus S, Leber A, Marcon MJ, Jordan JA, Martin JM, et al. A one-step, real-time PCR assay for rapid detection of rhinovirus. J Mol Diagn. 2010 Jan;12(1):102-8.

6. Kaida A, Kubo H, Takakura K, Sekiguchi J, Yamamoto SP, Kohdera U, et al. Associations between co-detected respiratory viruses in children with acute respiratory infections. Japanese journal of infectious diseases. 2014;67(6):469-75.

7. Kume Y, Hashimoto K, Shirato K, Norito S, Suwa R, Chishiki M, et al. Epidemiological and clinical characteristics of infections with seasonal human coronavirus and respiratory syncytial virus in hospitalized children immediately before the coronavirus disease 2019 pandemic. J Infect Chemother. 2022 Jul;28(7):859-65.

8. Dare RK, Fry AM, Chittaganpitch M, Sawanpanyalert P, Olsen SJ, Erdman DD. Human coronavirus infections in rural Thailand: a comprehensive study using real-time reverse-transcription polymerase chain reaction assays. J Infect

Dis. 2007 Nov 1;196(9):1321-8.

9. Owusu M, Annan A, Corman VM, Larbi R, Anti P, Drexler JF, et al. Human coronaviruses associated with upper respiratory tract infections in three rural areas of Ghana. PloS one. 2014;9(7):e99782.

10. Wong S, Pabbaraju K, Pang XL, Lee BE, Fox JD. Detection of a broad range of human adenoviruses in respiratory tract samples using a sensitive multiplex real-time PCR assay. J Med Virol. 2008 May;80(5):856-65.

11. WHO. WHO information for the molecular detection of influenza viruses. 2021 [cited 2022 14 July]; Available from: https://cdn.who.int/media/docs/default-source/influenza/molecular-detention-of-influenza-viruses/protocols_influenza_virus_detection_feb_2021.pdf?sfvrsn=df7d268a_5
